# Patterns and Variation in Benthic Biodiversity in a Large Marine Ecosystem

**DOI:** 10.1371/journal.pone.0135135

**Published:** 2015-08-26

**Authors:** Susan E. Piacenza, Allison K. Barner, Cassandra E. Benkwitt, Kate S. Boersma, Elizabeth B. Cerny-Chipman, Kurt E. Ingeman, Tye L. Kindinger, Jonathan D. Lee, Amy J. Lindsley, Jessica N. Reimer, Jennifer C. Rowe, Chenchen Shen, Kevin A. Thompson, Lindsey L. Thurman, Selina S. Heppell

**Affiliations:** 1 Department of Fisheries and Wildlife, Oregon State University, Corvallis, Oregon, United States of America; 2 Department of Integrative Biology, Oregon State University, Corvallis, Oregon, United States of America; 3 Department of Geographic Information Science, Oregon State University, Corvallis, Oregon, United States of America; Consiglio Nazionale delle Ricerche (CNR), ITALY

## Abstract

While there is a persistent inverse relationship between latitude and species diversity across many taxa and ecosystems, deviations from this norm offer an opportunity to understand the conditions that contribute to large-scale diversity patterns. Marine systems, in particular, provide such an opportunity, as marine diversity does not always follow a strict latitudinal gradient, perhaps because several hypothesized drivers of the latitudinal diversity gradient are uncorrelated in marine systems. We used a large scale public monitoring dataset collected over an eight year period to examine benthic marine faunal biodiversity patterns for the continental shelf (55–183 m depth) and slope habitats (184–1280 m depth) off the US West Coast (47°20′N—32°40′N). We specifically asked whether marine biodiversity followed a strict latitudinal gradient, and if these latitudinal patterns varied across depth, in different benthic substrates, and over ecological time scales. Further, we subdivided our study area into three smaller regions to test whether coast-wide patterns of biodiversity held at regional scales, where local oceanographic processes tend to influence community structure and function. Overall, we found complex patterns of biodiversity on both the coast-wide and regional scales that differed by taxonomic group. Importantly, marine biodiversity was not always highest at low latitudes. We found that latitude, depth, substrate, and year were all important descriptors of fish and invertebrate diversity. Invertebrate richness and taxonomic diversity were highest at high latitudes and in deeper waters. Fish richness also increased with latitude, but exhibited a hump-shaped relationship with depth, increasing with depth up to the continental shelf break, ~200 m depth, and then decreasing in deeper waters. We found relationships between fish taxonomic and functional diversity and latitude, depth, substrate, and time at the regional scale, but not at the coast-wide scale, suggesting that coast-wide patterns can obscure important correlates at smaller scales. Our study provides insight into complex diversity patterns of the deep water soft substrate benthic ecosystems off the US West Coast.

## Introduction

The existence of consistent, global patterns in biodiversity has long intrigued ecologists, as general patterns hint at common underlying mechanisms in the search for universal laws in ecology. A robust pattern observed across taxa and over large spatial scales is the inverse relationship between species biodiversity and latitude, with increasing diversity towards the equator (latitudinal diversity gradient; [1–3]). This gradient has largely been observed in terrestrial systems, with important contributions from the study of extinct marine taxa [4–6] and increasing interest in patterns of extant marine diversity [7–10]. The latitudinal diversity gradient is hypothesized to be caused by many different ecological and evolutionary processes that interact over space and time (see [11–13] for recent summaries). Given the challenge of inferring process from pattern, disentangling these hypothesized drivers remains one of the great challenges in macroecology [14]. Deviations from the latitudinal diversity gradient [15] provide an opportunity to examine what conditions “break the rule”. Marine systems, in particular, provide such an opportunity, given that marine diversity does not always follow a strict latitudinal gradient and two of the main hypothesized drivers of the latitudinal diversity gradient (i.e., high diversity in tropics results from high temperature and high productivity) are uncorrelated in some marine environments [16].

Notably, marine diversity often shows inconsistent patterns across latitude. Although a landmark analysis of ~200 studies found a strong latitudinal gradient in marine biodiversity, the pattern differed in strength among habitat types, organisms, and spatial scales [17]. Other studies have found inconsistent relationships with latitude when limited to a few taxa [18,19] or spatial scales smaller than an entire continent [20,21]. Such studies have found no relationship between marine diversity and latitude, or even a reverse relationship, i.e. higher diversity at higher latitudes, often the patterns are attributed to depth or other physical gradients (e.g., [22,23]). By explicitly examining and accounting for factors that contribute to deviations in marine latitudinal diversity gradients, we can gain insight into the processes that drive biodiversity patterns in marine systems.

Patterns of marine diversity might not follow a strict latitudinal gradient because the drivers of marine diversity themselves are not always correlated with latitude. In terrestrial systems, latitude and elevation are proxies for gradients in temperature, divergence time and evolutionary stability that drive the gradient in diversity [6,24–26]. In marine systems, depth acts as a similar proxy to latitude, as increasing depth co-varies with decreasing light availability, decreasing temperature, increasing pressure, decreasing productivity and greater stability in salinity and temperature [27]. Further, depth may be a more influential driver at smaller spatial scales, just as elevational gradients strongly influence terrestrial diversity [8,28]. This pattern has been observed in groundfish assemblages in the North Pacific, where depth is a stronger predictor of diversity than latitude [29,30]. At greater depths, benthic organisms are often tightly associated with particular substrates, such as sand, mud, and rock, which can influence their broader distributional patterns. In particular, benthic marine faunal diversity is often highest around rocky outcroppings, where habitat complexity is high, leading to more physical space for organisms [31,32]. Thus, in marine systems, if substrate type varies latitudinally, this may act as another important driver of marine biodiversity.

Deviations from the latitudinal gradient in diversity may also result from the implicit time-averaged nature of most biodiversity studies. Macroecological patterns of biodiversity have largely been evaluated using long-term time series or data from a snapshot in time [33], but diversity is unlikely to be spatially consistent from year-to-year, particularly in highly dynamic systems that include species with broad-scale dispersal [34]. In marine systems, the environmental and climatological factors that play an important role in structuring communities vary inter-annually (e.g. El Niño Southern Oscillation and the Pacific Decadal Oscillation; [35–37]). Thus, explicitly evaluating inter-annual variation in diversity on ecological timescales could explain deviations from traditional latitudinal gradients and uncover the underlying processes that shape marine biodiversity.

Given the difficulty in disentangling these underlying drivers of marine biodiversity, ecologists have turned to comparisons of multiple metrics of biodiversity to help tease apart *a priori* hypotheses about the different drivers [38–41]. While traditional metrics of biodiversity focus on taxonomic distinctions among species, analysis of functional diversity is a way to understand the mechanisms underlying the distribution of organisms by relating functional traits to environmental drivers [41,42]. In particular, by describing the variation in species functional traits, functional diversity captures patterns of biodiversity that species richness and abundance do not. Examination of broad-scale latitudinal patterns in functional diversity and the relationships among different metrics of biodiversity offer a way forward towards quantifying biodiversity gradients and understanding their drivers [15,41,43–47].

We tested for the presence of a latitudinal diversity gradient in temperate marine communities with a large-scale, spatio-temporal analysis of patterns in marine benthic faunal diversity. Because patterns of marine biodiversity across latitude are sensitive to a myriad of environmental drivers, temporal variability, and the biodiversity metric used, these factors must be accounted for in any analysis of latitudinal gradients. Thus, using a public scientific monitoring dataset spanning the California Current Large Marine Ecosystem off the US West Coast, we asked, 1) Is there a latitudinal gradient in marine faunal diversity? 2) What is the role of depth and substrate in structuring such a latitudinal gradient? and 3) Are these patterns dependent on space and time? We hypothesized that marine benthic diversity will be higher at lower latitudes [17], in deeper waters [16], and around rocky substrates [31,32]. Additionally, we hypothesized that these patterns will be sensitive to inter-annual variability in the coastal environment, though *a priori* we did not have an expectation about the direction of the impact on marine benthic diversity patterns. Following Hillebrand [3,17], we expected that the strength of the latitudinal diversity gradient will be stronger at coast-wide scales than at smaller, regional scales, where oceanographic processes tend to influence community structure and function [48–50]. To test these hypotheses, we examined spatio-temporal patterns of diversity for benthic fish and invertebrate species across eight years using three diversity metrics (taxonomic richness, taxonomic diversity, and functional diversity). Given the importance of biodiversity to maintaining ecosystem function and the gap in knowledge of biodiversity patterns in the California Current marine system, understanding the variability of species and functional trait distributions over large spatial scales is essential to conserving the structure and function of this and other systems in the face of environmental change.

## Methods

### Study Area

**Fig 1:**
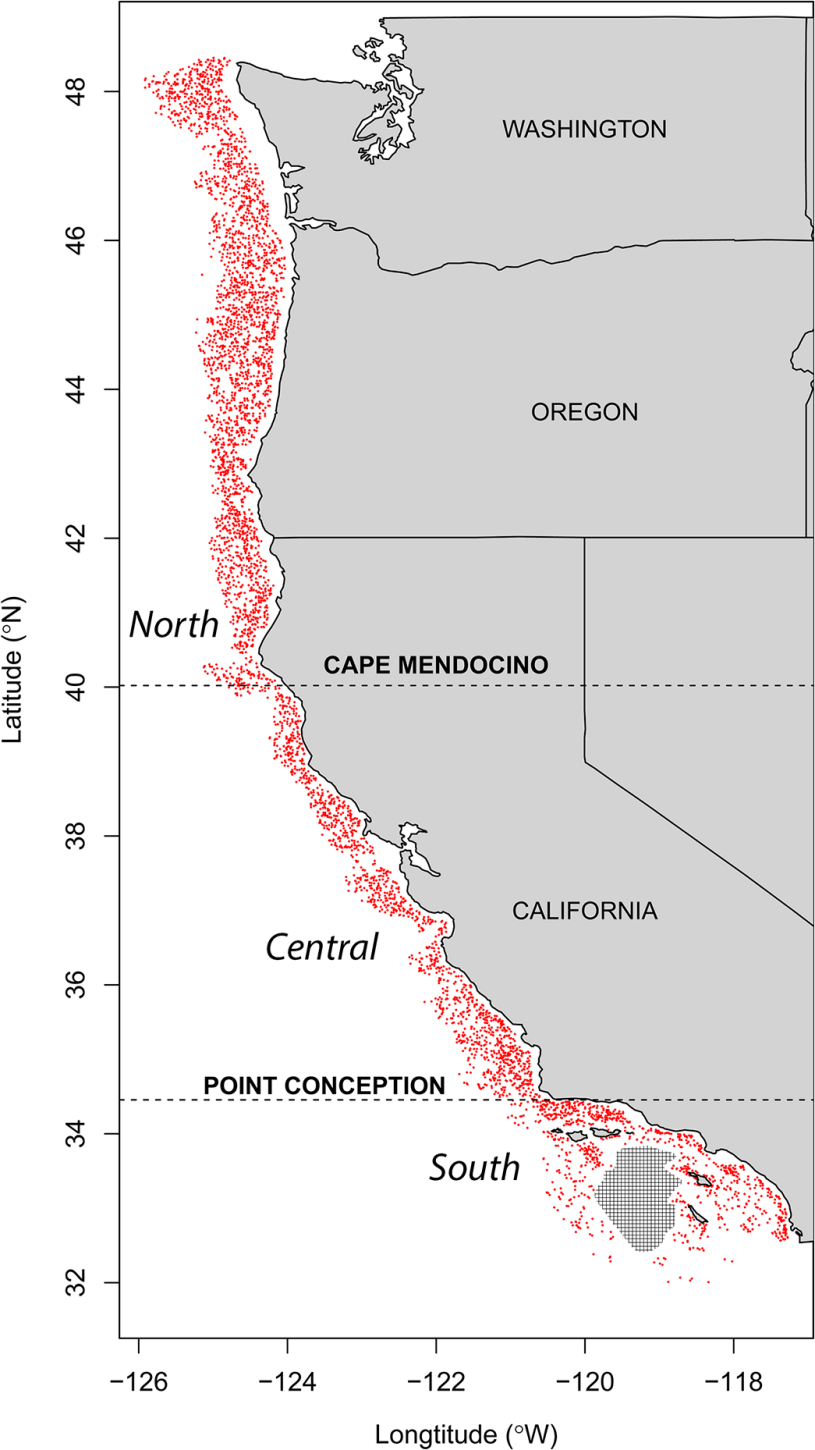
Map of Northeast Pacific Ocean Trawl Survey. Red dots indicate location of the West Coast Groundfish Bottom Trawl Survey (WCGBTS) trawls from 2003–2010. Cape Mendocino and Point Conception break the study area into the three biogeographic regions used in the regional analyses (North of Cape Mendocino, Central, and South of Point Conception). The WCGBTS is unable to sample in the area within the hashed lines. The map was drawn using the *maps* package for R.

The California Current Large Marine Ecosystem (CCLME) comprises the coastal and offshore region south of British Columbia to the southern tip of Baja California, encompassing 2.2 million km^2^ surface area (Fig 1; [51,52]). The CCLME spans temperate to subtropical climatic regions and experiences strong seasonal upwelling, which brings cold, nutrient rich water to the surface during the summer and increases productivity. The benthic habitat includes hard substrate (submarine canyons, ridges, and rocky reefs) within an expansive matrix of unconsolidated sand, mud, and gravel [53]. Loose sediment particles range from silt to boulders. The continental shelf is fairly narrow, so the continental slope (approximately >200m depth) is relatively close to the coast, with coastal proximity to deep slope greatest in the south [52]. High inter-annual variability in temperature and productivity characterizes the CCLME. During the period of data collection, summer bottom temperatures in the study area ranged from 2.91°C–14.7°C.

### Species Abundance Data

We used fish and invertebrate data from the West Coast Groundfish Bottom Trawl Survey (WCGBTS) conducted by the Northwest Fisheries Science Center (NWFSC), National Oceanic and Atmospheric Administration (NOAA), US [54]. WCGBTS is a fishery-independent survey led by NOAA scientists and conducted annually in conjunction with chartered commercial fishing vessels to collect and identify species of fish and invertebrate taxa. We examined data for the years 2003–2010, as 2003 was the first year of data collection by the NWFSC and 2010 was the most recent year of data available. Scientific survey trawls are conducted annually from mid-May until late October. The survey follows a stratified-random sampling design based on geographic location (north or south of Point Conception, California) and depth (55–183 m, 184–549 m, or 550–1,280 m). The survey samples both the continental shelf (depth 55–183 m) and the continental slope (depth 184–1,280 m) from Cape Flattery, WA (47°20′N) to the US-Mexico border (32°40′N). Tows employed a Aberdeen-type net, with a small mesh (3.81cm^2^) codend liner to retain smaller fish and invertebrates [54]. Trawling occurred over soft to rocky benthic substrate within randomly selected cells (1.5 nautical miles (nm) longitude by 2.0 nm latitude) in the three depth strata. Additional information on the survey methodology can be found in Bradburn et al. [54].

For 2003–2010, the dataset was comprised of 5,162 trawls. The number of trawls per year has increased since 2003, ranging from 497 (in 2004) to 716 (in 2010). Because of the underlying increase in sampling effort over time, we used species accumulation curves to determine whether sampling had been sufficient in each year (S1 File). Species accumulation curves for all years approached asymptotes, indicating that the data did not require rarefaction and that sampling was sufficient (S3 Fig). However, invertebrates were identified to the species level less frequently in 2003 than in subsequent years (K. Bosley, personal communication) and reached a lower asymptote than all other years. For this reason, we restricted analysis of invertebrates to 2004–2010.

The WCGBTS was designed to provide information on biomass, abundance, length, and age of commercially harvested fish for population assessments, but all individuals captured (regardless of commercial interest) are identified to the lowest taxonomic level possible (S1 Table). Species that are unidentified onboard were labeled, frozen, or preserved in formalin and saved for later identification [54]. Due to time constraints between tows, staff did not enumerate individual organisms; instead, the biomass of each identified taxon was determined on deck. We standardized the biomass of each species to the area swept of the trawl net (kg/ha), a measure of catch per unit effort (CPUE), to control for differences in the sampling area across trawls. We minimized the risk of double counting species by combining weights of species rarely identified to species level into a single category by genus (S1 File). We also eliminated pelagic species to avoid inflating diversity estimates with species incidentally caught during net deployment or retrieval.

### Environmental Data

We included three other predictors known to influence diversity that co-vary with latitude: depth, year, and substrate. Depth was recorded at the mid-point of each trawl. Bottom temperature was also recorded for each trawl, but it was strongly collinear with depth. We chose to use depth as a predictor instead of temperature, because depth captures additional dependent factors beyond temperature. Year was included as a predictor not only as a proxy for unmeasured environmental conditions, but also to account for the temporal structure of the data collection across eight years.

We extracted seafloor substrate type from the Surficial Geologic Habitat Map (SGHM) GIS layer for the US West Coast [55]. The SGHM combines data collected from bathymetry, field-sampled point surveys, acoustic imagery, and sub-bottom analysis to create a mixed-resolution map of the seafloor, interpreted using the highest level of detail that could be justified through each method. Thus, habitat identification represented on the map is accurate, but varies in precision (C. Romsos, pers. communication). We used the SGHM to create a binary variable classifying the sea-floor bottom as either soft (silt, mud, sand, and gravel) or rocky (cobbles to small boulders). We then overlaid trawl survey points onto the habitat layer and sampled the substrate type at the point of the trawl. Each point was assigned the primary habitat type over which the trawl was towed, thus mixed-habitat trawls are not represented in this dataset.

### Biodiversity Measures

We examined two taxonomic biodiversity indices, species richness (S), and Shannon diversity (H′) for fish and invertebrates. We calculated H′ as the sum of proportional biomass/area swept (kg/ha) of all species in a sample:

$$H'=\sum_{i=1}^{n}p_{\text{i}}\;\text{ln}\;p_{\text{i}}$$ (1)

where *p*_*i*_ is the proportion of biomass (CPUE) of species *i* in a trawl [56]. We chose this measure for two reasons: our data were measured as biomass, rather than counts of individuals/species, which precluded the use of many biodiversity indices; and H′, in which biomass units have been applied as a measure of abundance [57–61], is a commonly used and readily comparable index of biodiversity [61,62].

In addition, we used a third measure, Rao’s quadratic entropy index (Q) to quantify functional diversity [63], or the diversity of traits in a system, using life history and trophic characteristics of the species observed. We only calculated Q measures for fish, because limited data on invertebrate traits were available. For fishes, we acquired information on a total of 18 traits using FishBase (S1 File and S2 Table; [64,65]). We used these traits to calculate Q for each trawl. Q is a measure of the abundance-weighted trait diversity of a sample:

$$Q=\sum_{i=1}^{S-1}\cdot \sum_{j=i+1}^{S}d_{ij}p_{i}p_{j}$$ (2)

where *S* is the total species richness, *d*_*ij*_ is the dissimilarity in traits between species *i* and species *j*, and *p*_*i*_ and *p*_*j*_ are the relative abundances of species *i* and *j*. We calculated Q because trait diversity may show patterns that are not otherwise seen using measures of taxonomic richness and diversity [42,66].

### Statistical Analysis

We used model-averaging to fit a series of generalized linear models (GLMs) to test our hypotheses regarding fish and invertebrate biodiversity gradients in relation to latitude and environmental co-variates for the five response variables: S and H′ for fish and invertebrates, and Q for fish species only. This approach is common in the ecological literature for its ability to flexibly account for many environmental co-variates and differences in the distribution of response variables while preserving the interpretability of a linear framework [67]. All GLMs included the same four potential explanatory variables (latitude, year of the trawl, depth, and substrate type) based on previous work done in this system, as well as studies using fishery survey data to describe abundance patterns [29,30,68]. We restricted the candidate set to linear models 1) to retain interpretability and facilitate comparison with previous tests of a latitudinal diversity gradient, 2) because processes hypothesized to cause a latitudinal diversity gradient provide no mechanistic framework for determining *a priori* the appropriate non-linear functional form, and 3) because we sought to keep our methods consistent across predictors. We then used a model averaging information theoretic approach in conjunction with the GLMs [69,70]. This approach tests biological hypotheses in the form of candidate models and provides a quantitative measure of relative support for competing hypotheses based on a robust statistical framework [71]. Model selection is preferred to traditional null hypothesis testing for observational data, where causative factors cannot be isolated by sampling design or when multiple models have similar levels of support [71–73]. Traditional, stepwise model selection routines have been shown to lead to spurious conclusions, as inference is conditional on the single “best” model and variables included [74]. In contrast, Akaike’s Information Criterion (AIC)-based model averaging incorporates the inherent uncertainty in model selection and thus improves parameter estimation when competing models have similar support from the data, and protects against spurious conclusions regarding important variables in the observed system [75].

We treated each combination of maximum likelihood-estimated parameters in the regression models as competing hypotheses describing the interaction between the explanatory variables (latitude, depth, year and substrate) and S, H′, and Q, respectively. We considered the model (out of a possible 2^13^ = 8,192 models given by the number of environmental variables and their interactions) with the lowest AICc value the top model and used to calculate ∆AICc for each subsequent model ranked by AICc, where the best model’s ∆AICc = 0. We then calculated Akaike weights, *w*_*i*_, for each model [69,76]. We considered all models with a ∆AICc < 2 to be supported by the data and therefore included them, creating a confidence set of top models [69,76]. From the confidence set of models, we calculated model averaged parameter estimates (β_i_) as weighted sums of parameter coefficients given by the product of the parameter estimate (β_i_) in model *i* times its Akaike weight, *w*_*i*_ [76]. Similarly, we calculated 95% confidence intervals (95% CI) around the model averaged parameters from the confidence set of models [69,76]. We inferred that variables with confidence intervals which did not include zero were related to the biodiversity metric being modeled. We then used the sign of the model averaged parameters to infer the direction of the relationship of the variables correlated with biodiversity in the CCLME for both individual variables as well as interactions among them.

The use of generalized linear models in this framework allowed us to account for differences in the functional form of our response variables. Fish and invertebrate H′ and Fish Q fit the assumptions of normality, and thus were modeled with a normal distribution. We modeled invertebrate S with a negative binomial distribution, rather than the more commonly used Poisson distribution, because diagnostic plots of the average square residuals to the mean revealed overdispersion [77]. We modeled fish S with Poisson because although the fish S data were estimated to be slightly overdispersed (Ψ = 1.099), a negative binomial model did not provide a better fit to the data than a Poisson model (Likelihood ratio test, χ2 = 1.714, p = 0.1905). We examined histograms of residuals for all models and used variance inflation factors to determine that all predictors were non-collinear. We checked all responses (S, H′, and Q) for significant outliers using leverage scores and studentized residuals and found no points with both high leverage and high residuals. We also analyzed the residuals of our best models for spatial autocorrelation using Moran’s I. Values fell near zero, indicating that models adequately accounted for spatial autocorrelation without further correlation structures (see S1 File). We calculated R^2^ (for H′ and Q) and McFadden’s pseudo-R^2^ (for S) for all models. Calculating R^2^ wasn’t possible for our final averaged models, so we calculated R^2^ using a model that included all the (un-averaged) parameters selected for in the final model. Thus, these R^2^ and pseudo-R^2^ estimates should be interpreted with caution. All analyses were conducted in R, version 2.12.0, with spatial patterns of biodiversity displayed using the *Fields* and *Maps* packages, and model selection from the *MuMIn* package [78,79].

### Comparisons to Models Accounting for Latitude

To test whether latitude is an important driver of marine biodiversity, we used the evidence ratio of the AICc-selected best model with and without latitude (Eq 3). The ratio, ρ, provides an estimate of the relative support in the data for two competing models based on the AICc weights [76]:

$$\rho =w_{i}/w_{j}$$ (3)

where *w*_*i*_ and *w*_*j*_ are the AICc weights of models *i* and *j*. We compared the top-ranking model that included latitude to the top-ranking model that did not include latitude for all five responses. This metric allowed us to determine, given the data, the factor by which the complex best model is potentially more likely compared to a model without latitude.

### Comparisons Across Biogeographic Regions

Previously published research suggests that there may be discrete regions in the California Current system in regard to biodiversity and that the factors affecting biodiversity patterns within regions may vary [80]. To elucidate patterns at the regional scale that may be obscured by our coastwide analysis, we divided the coastline into three biogeographic regions, north of Cape Mendocino, CA (North), between Cape Mendocino and Point Conception, CA (Central), and south of Point Conception (South; Fig 1). We performed the same GLM/model selection routines for these three regions as we did for the entire CCLME dataset. With the addition of these three regional models for each of our three diversity responses to those of the entire system, we carried out 20 total model selection procedures (four total spatial datasets five diversity responses) in this study.

## Results

**Fig 2:**
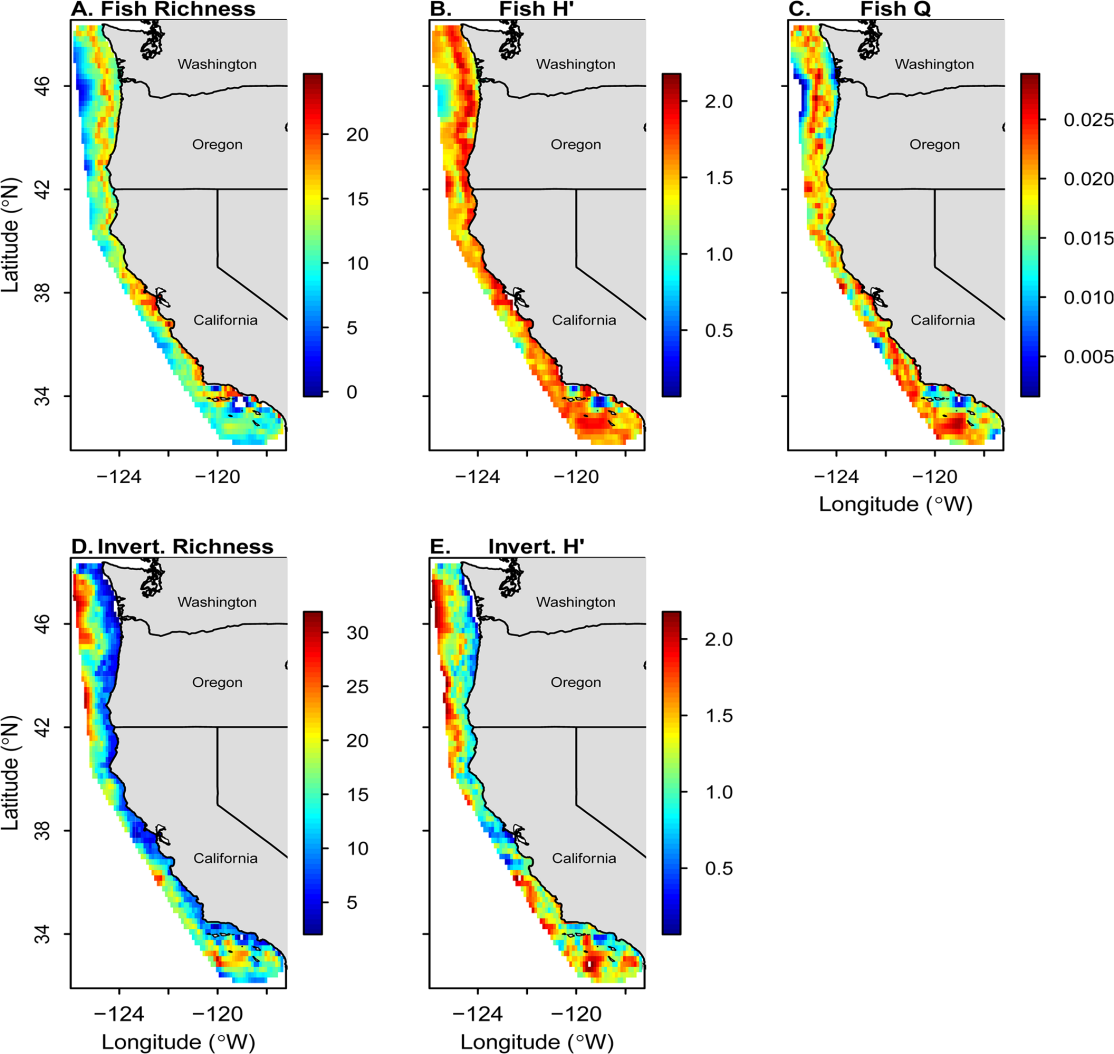
Thin-plate spline interpolation of fish species richness (A), fish Shannon diversity (B), fish functional diversity (C), invertebrate species richness (D) and invertebrate Shannon diversity (E) from the West Coast Groundfish Bottom Trawl Survey, 2003–2010 (fish) and 2004–2010 (invertebrates). Warmer (red) areas indicate regions of higher biodiversity, while cooler (blue) regions indicate lower biodiversity. The maps were drawn using the *maps* package for R.

**Fig 3:**
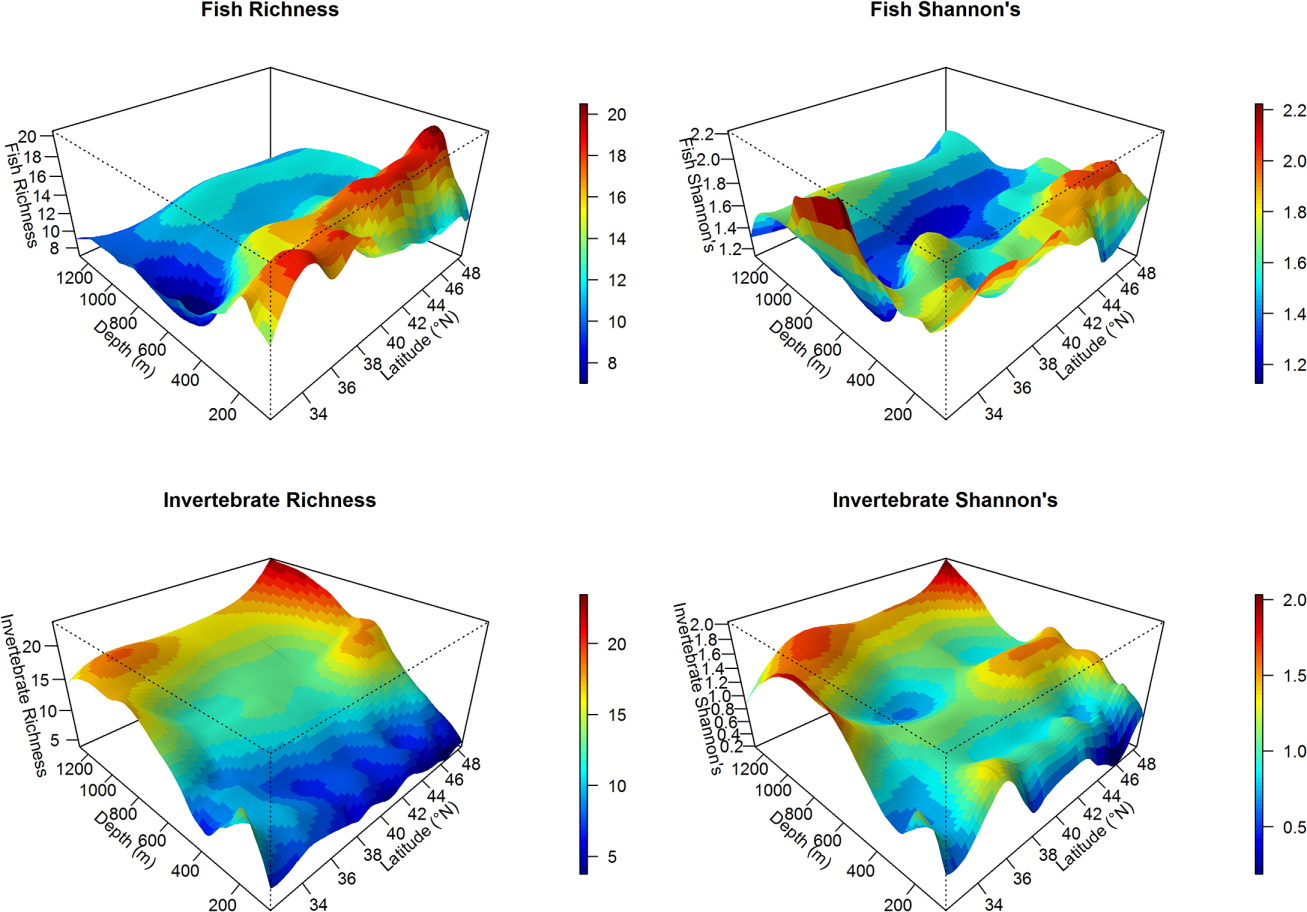
Relationships among depth and latitude and (A) fish species richness, (B) fish Shannon diversity, (C) invertebrate species richness, and (D) invertebrate Shannon diversity. Data were visualized using a smoothing function to examine the interactive effect of depth and latitude on species diversity. Across latitude, fish diversity generally decreases after about 200 m depth, while invertebrate diversity increases with depth. However for both taxa, abundance-weighted diversity (Shannon’s, B, D) shows greater heterogeneity across latitude and depth.

**Fig 4:**
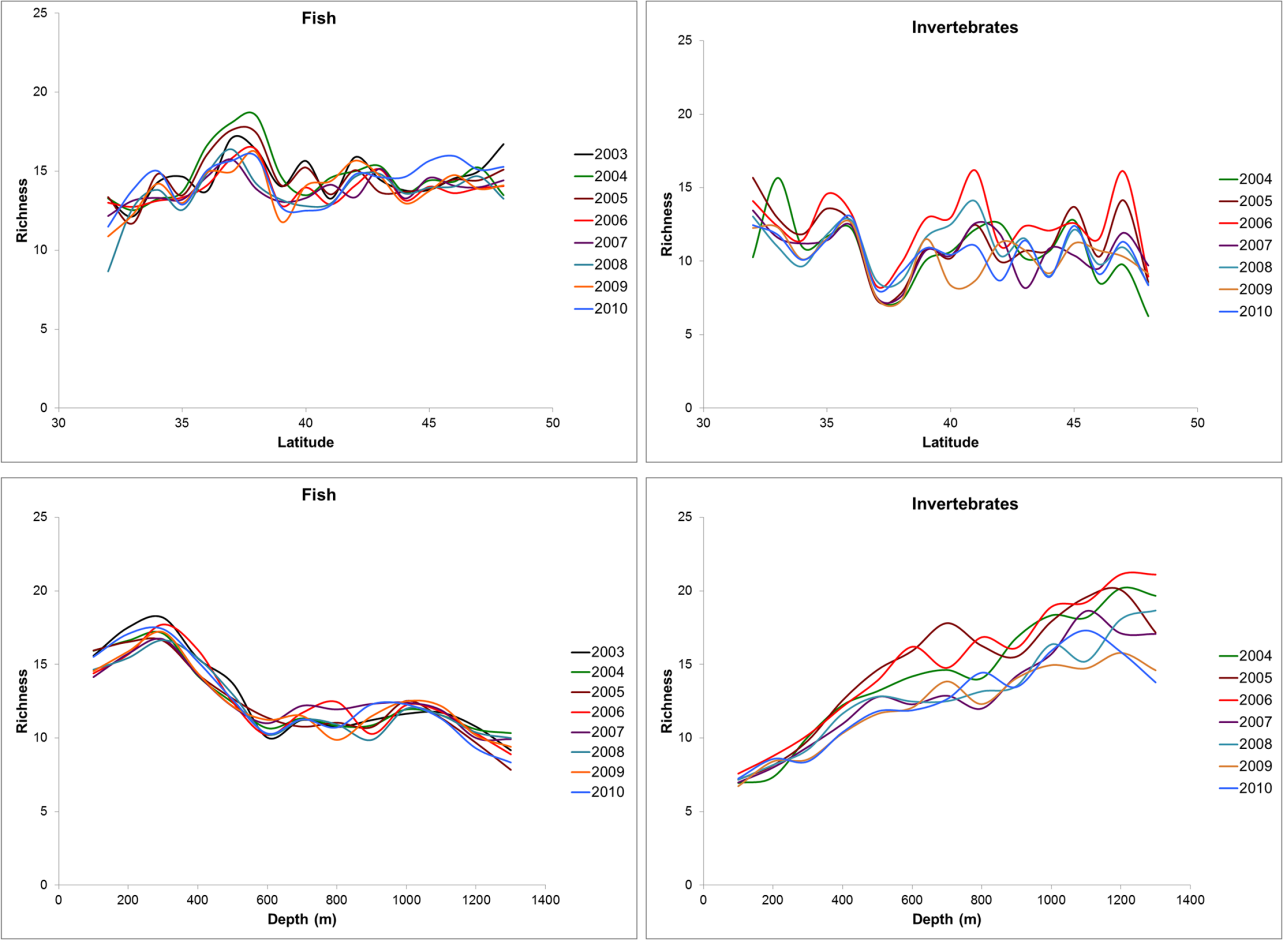
Mean fish and invertebrate species richness by degree latitude (A and C) and depth (B and D) for 2003–2010 (fish) and 2004–2010 (invertebrates) from the West Coast Groundfish Bottom Trawl Survey. Species richness by latitude was calculated by averaging trawl results over 1 degree bins, while richness by depth was calculated by averaging richness values within a 100-meter depth bin. Error bars have been left off for visualization of temporal variability in the averages.

Overall, the trawl survey encountered 233 fish and 310 invertebrate taxa in 171 families across all years, from Washington to California (S1 Table). Maps of richness (S), Shannon diversity (H′), and functional diversity (Q) suggested that spatial patterns across both depth and latitude differ between fish and invertebrate taxa (Figs 2 and 3). The relationships of fish and invertebrate S with latitude and depth over time revealed some large scale patterns and temporal variability (Fig 4, S1 and S2 Figs). We observed highly consistent patterns across years in the relationship between depth and fish S, but more variability among years in latitude and fish S and invertebrate S (Fig 4).

### Patterns Across the CCLME

Latitude represented an important driver of all metrics of diversity for fish and invertebrates across the entire spatial region of the CCLME, as all AICc-selected models with latitude were orders of magnitude more likely than those without latitude (all ρ ≥ 131.35, Table 1). While latitude appeared in all top-ranking models, its relationship with the different metrics of diversity varied and was dependent on depth, year, and substrate. In other words, diversity was not always highest at low latitudes, but instead showed complex patterns in space and time.

**Table 1:** The effect of latitude on model importance for fish and invertebrate richness (S), Shannon diversity (H′) and Rao’s quadratic entropy (Q).

	Fish S	Fish H′	Fish Q	Invert S	Invert H′
**AICc weight for best model with Latitude, w**_**i**_	0.1468	0.0518	0.0525	0.2481	0.1625
**AICc weight for best model without Latitude, w**_**j**_	1.98∙10^−52^	3.94∙10^−4^	1.64∙10^−17^	1.23∙10^−13^	6.67∙10^−6^
**Evidence Ratio, ρ (w**_**i**_ **/w**_**j**_ **)**	7.52∙10^50^	131.35	3.20∙10^15^	2.018∙10^12^	24362.82

This was determined using the evidence ratio (ρ), which is calculated by first determining the best model using Akaike’s Information Criterion correction (AICc), and then dividing the AICc weight of the model when the term is in the model (w_i_) by the AICc weight of the model when the term is removed (w_j_). The greater the ρ, the more important latitude is as a predictor in the model.

Overall, fish S increased with increasing latitude, but this relationship also depended on the effects of depth, year, and substrate (Table 2, Fig 3 and S1 Fig). For example, the effect of latitude on fish S was stronger in rocky areas than in soft-sediment areas (Table 2). Furthermore, fish S appeared to be more influenced by depth than latitude, with a peak in richness at approximately 200 m and then declined sharply with increasing depth across most latitudes (Figs 3 and 4). Although there were obvious peaks in fish H′ and Q at certain depth and latitude combinations (Figs 2 and 3), there were no significant predictors for fish H′ or Q, indicating that these responses did not vary linearly based on our explanatory variables (Table 2). Fish H′ and Q did not change across the latitudinal gradient, suggesting that our explanatory variables did not AFFECt these abundance-weighted metrics (Table 2).

**Table 2:** Generalized linear model-averaged results for fish richness (S), Shannon diversity (H′) and Rao’s quadratic entropy (Q).

Metric (Adj.R^2^)	Variable	Estimate	Adjusted SE	Lower CI	Upper CI	RI
**S** (0.053 pseudo)	(Intercept)	-0.6203	38.681	-76.434	75.193	.
	Depth	0.0382	0.1013	-0.1603	0.2366	1.00
	Latitude	0.4794	0.9361	-1.3554	2.3142	1.00
	Substrate	-0.6367	4.9834	-10.4039	9.1305	1.00
	Year	0.0020	0.0193	-0.0358	0.0397	1.00
	Depth∙Latitude	-0.0016	0.0025	-0.0065	0.0032	1.00
	Depth∙Substrate	3.72∙10^−5^	0.0003	-0.0006	0.0007	1.00
	Depth∙Year	-2.00∙10^−5^	0.0001	-0.0001	0.0001	1.00
	**Lat∙Substrate**	**0.0101**	**0.0041**	**0.0021**	**0.0181**	**1.00**
	Latitude∙year	-0.0005	0.0006	-0.0016	0.0006	0.49
	Substrate∙Year	0.0009	0.0077	-0.0143	0.0160	0.10
	**Depth∙Latitude∙Year**	**2.12∙10** ^**−6**^	**1.08∙10** ^**−6**^	**2.44∙10** ^**−9**^	**4.24∙10** ^**−6**^	**0.39**
	Depth∙Latitude∙Substrate	1.13∙10^−5^	1.51∙10^−5^	-1.82∙10^−5^	4.09∙10^−5^	0.26
**H′** (0.079)	(Intercept)	50.029	53.460	-54.751	154.81	.
	Depth	-0.0622	0.0985	-0.2552	0.1308	1.00
	Latitude	-0.5496	1.2775	-3.0535	1.9543	1.00
	Substrate	-0.3256	0.2863	-0.8867	0.2355	0.45
	Year	-0.0242	0.0266	-0.0764	0.0281	1.00
	Depth∙Latitude	0.0011	0.0026	-0.0039	0.0062	0.82
	Depth∙Substrate	0.0003	0.0005	-0.0007	0.0013	0.39
	Depth∙Year	3.45∙10^−5^	0.0001	-0.0001	0.0001	0.89
	Latitude∙Substate	0.0099	0.0064	-0.0027	0.0224	0.33
	Latitude∙Year	0.0011	0.0008	-0.0006	0.0027	0.26
	Depth∙Latitude∙Year	-2.55∙10^−6^	1.51∙10^−6^	-5.51∙10^−6^	4.18∙10^−7^	0.18
	Depth∙Latitude∙Substrate	-2.49∙10^−5^	2.26∙10^−5^	-0.0001	1.94∙10^−5^	0.07
**Q** (0.064)	**(Intercept)**	**0.5818**	**0.2949**	**0.0038**	**1.1598**	.
	Depth	-0.0001	0.0002	-0.0006	0.0003	1.00
	Latitude	0.0014	0.0064	-0.0112	0.0140	1.00
	Substrate	-0.1351	0.2826	-0.6889	0.4188	1.00
	Year	-0.0003	0.0001	-0.0006	3.90∙10^−6^	1.00
	Depth∙Latitude	8.86∙10^−8^	5.53∙10^−8^	-1.98∙10^−8^	1.97∙10^−7^	0.69
	Depth∙Substrate	7.90∙10^−6^	9.78∙10^−6^	-1.13∙10^−5^	2.71∙10^−5^	1.00
	Depth∙Year	1.65∙10^−7^	1.21∙10^−7^	-7.21∙10^−8^	4.02∙10^−7^	0.45
	Latitude∙Substrate	0.0002	0.0001	-0.0001	0.0004	0.38
	Latitude∙Year	-5.43∙10^−6^	7.99∙10^−6^	-2.11∙10^−5^	1.02∙10^−5^	0.11
	Substrate∙Year	0.0002	0.0002	-0.0002	0.0006	0.33
	Depth∙Latitude∙Substrate	-5.46∙10^−7^	3.60∙10^−7^	-1.25∙10^−6^	1.60∙10^−7^	0.18

The Akaike Information Criterion correction (AICc) was used to rank models and any model that ranked <2 ΔAICc was averaged to obtain final estimates presented. Bolded model terms have a 95% confidence interval (CI) that did not include zero. Relative importance (RI) refers to the proportion of output models that contained the term before the model estimates were averaged.

Consistent with the results of fish S, both invertebrate S and H′ were influenced by a positive interaction among latitude, depth, and year (Table 3), thus invertebrate S and H′ increased at higher latitudes, but also in deeper waters and through time. The clear positive relationship with depth is evident when invertebrate S and H′ is plotted against both depth and latitude (Fig 3). For both invertebrate S and H′, the effect of latitude was stronger in rocky substrates than soft substrates (Table 3). We did not examine Q for invertebrates due to lack of biological trait data.

**Table 3:** Generalized linear model-averaged results for invertebrate richness (S), and Shannon diversity (H′).

Metric (Adj.R^2^)	Variable	Estimate	Adjusted SE	Lower CI	Upper CI	RI
**S** (0.078 pseudo)	**(Intercept)**	**-259.49**	**81.892**	**-419.99**	**-98.986**	.
	**Depth**	**0.6201**	**0.1426**	**0.3406**	**0.8995**	**1.00**
	**Latitude**	**6.2038**	**1.9899**	**2.3037**	**10.1038**	**1.00**
	Substrate	0.2176	27.6184	-53.9134	54.3487	1.00
	**Year**	**0.1304**	**0.0408**	**0.0504**	**0.2104**	**1.00**
	**Depth∙Latitude**	**-0.0127**	**0.0035**	**-0.0196**	**-0.0058**	**1.00**
	Depth∙Substrate	-0.0545	0.0838	-0.2188	0.1098	0.37
	**Depth∙Year**	**-0.0003**	**0.0001**	**-0.0004**	**-0.0002**	**1.00**
	**Latitude∙Substrate**	**0.0192**	**0.0050**	**0.0094**	**0.0290**	**1.00**
	**Latitude∙Year**	**-0.0031**	**0.0010**	**-0.0050**	**-0.0012**	**1.00**
	Substrate∙Year	-0.0011	0.0227	-0.0455	0.0434	0.37
	Depth∙Substrate∙Year	0.0001	4.11∙10^−5^	-1.51∙10^−5^	0.0001	0.15
	**Depth∙Latitude∙Year**	**6.33∙10** ^**−6**^	**1.76∙10** ^**−6**^	**2.88∙10** ^**−6**^	**9.79∙10** ^**−6**^	**1.00**
**H′** (0.088)	**(Intercept)**	**-262.94**	**97.408**	**-453.86**	**-72.032**	.
	Depth	0.3648	0.1866	-0.0008	0.7304	1.00
	**Latitude**	**6.4989**	**2.3671**	**1.8594**	**11.138**	**1.00**
	**Substrate**	**-1.5722**	**0.4662**	**-2.4859**	**-0.6584**	**1.00**
	**Year**	**0.1315**	**0.0485**	**0.0364**	**0.2266**	**1.00**
	**Depth∙Latitude**	**-0.0102**	**0.0047**	**-0.0194**	**-0.0011**	**1.00**
	Depth∙Substrate	0.0015	0.0013	-0.0010	0.0041	1.00
	Depth∙Year	-0.0002	0.0001	-0.0004	0.0000	1.00
	**Latitude∙Substrate**	**0.0399**	**0.0121**	**0.0161**	**0.0637**	**1.00**
	**Latitude∙Year**	**-0.0032**	**0.0012**	**-0.0056**	**-0.0009**	**1.00**
	**Depth∙Latitude∙Year**	**5.11∙10** ^**−6**^	**2.32∙10** ^**−6**^	**5.62∙10** ^**−7**^	**9.65∙10** ^**−6**^	**1.00**
	Depth∙Latitude∙Substrate	-0.0001	3.12∙10^−5^	-0.0001	6.95∙10^−6^	0.62

The Akaike Information Criterion correction (AICc) was used to rank models and any model that ranked <2 ΔAICc was averaged to obtain final estimates presented. Bolded model terms have a 95% confidence interval (CI) that did not include zero. Relative importance (RI) refers to the proportion of output models that contained the term before the model estimates were averaged.

### Patterns Across Biogeographic Regions

When we divided the spatial extent of the survey into three biogeographic regions, we did not see a consistent weakening of the effect of latitudinal gradients, but instead the smaller-scale models revealed patterns that were obscured at the coast-wide scale (Table 4 and see S3 Table for full results). For example, fish H′, which was not associated with any co-variates at the coast-wide scale, was positively associated with the interaction of latitude, depth, and year in the South region (Table 4 and S3 Table). However, no model co-variates strongly explained the patterns of fish H′ in the North and Central regions. Likewise, several important predictors for fish Q emerged at the regional scale, in contrast to the coast-wide analysis, which did not find any significant predictors. Fish Q was significantly affected by latitude in all three regions, but the specific interactions of latitude and the other predictors differed among regions. In the North region, the interaction between latitude, depth, and year was positive (Table 4 and S3 Table). In the Central region, when the substrate was rocky, fish Q decreased with latitude (Table 4 and S3 Table). In the South region, there was a greater effect of latitude at shallower depths, and a greater effect of depth in rocky substrates (Table 4 and S3 Table).

**Table 4:** Summary of model-averaged variables included in the confidence model set for fish richness (S), Shannon diversity (H′) and Rao’s quadratic entropy (Q) and invertebrate S and H′ for coast-wide and the three biogeographic regions, North of Cape Mendocino, the Central Region, and South of Point Conception.

Taxa Metric	Adj. R^2^	Scale	Variables in Confidence Set (which have 95% CIs that do not include zero)
**Fish S**	0.053 (pseudo)	Coast	-intercept + latitude∙substrate + depth∙latitude·year
	0.024 (pseudo)	North	depth∙year–latitude∙substrate·year
	0.075 (pseudo)	Central	latitude·substrate
	0.167 (pseudo)	South	depth–depth∙latitude–depth∙substrate–depth∙year + substrate·year + depth·latitude·substrate + depth∙latitude·year–latitude∙substrate·year
**Fish H′**	0.079	Coast	All variables have 95% CIs that include zero
	0.110	North	All variables have 95% CIs that include zero
	0.072	Central	All variables have 95% CIs that include zero
	0.165	South	-depth∙year + depth∙latitude·year
**Fish Q**	0.064	Coast	intercept
	0.071	North	-depth∙year + latitude·substrate + depth∙latitude·year
	0.072	Central	latitude∙substrate
	0.066	South	- depth∙latitude + depth∙substrate
**Invertebrates S**	0.078 (pseudo)	Coast	-intercept + depth + latitude + year–depth∙latitude–depth∙year + latitude·substrate–latitude·year + depth∙latitude·year
	0.086 (pseudo)	North	intercept–latitude–year + depth∙latitude–depth∙substrate–latitude·substrate + latitude·year + depth∙latitude·substrate
	0.094 (pseudo)	Central	depth–depth∙year–depth∙latitude·substrate
	0.062 (pseudo)	South	depth–depth∙latitude–depth∙year + depth∙substrate·year
**Invertebrates H′**	0.088	Coast	-intercept + latitude–substrate + year–depth∙latitude + latitude·substrate–latitude·year + depth∙latitude·year
	0.104	North	All variables have 95% CIs that include zero
	0.132	Central	-intercept + latitude + year–depth∙latitude–depth∙year–latitude·year + depth∙latitude·substrate + depth∙latitude·year + latitude·substrate·year–depth∙latitude·substrate·year
	0.169	South	depth·substrate

The Akaike Information Criterion correction (AICc) was used to rank models and any model that ranked <2 ΔAICc was averaged to obtain final estimates. Only model terms which have a 95% confidence interval (CI) that did not include zero are included. Complete model averaged results for the biogeographic regions are presented in S3 Table.

Within each biogeographic region, all metrics of diversity were related to latitude, but, as on the whole-coast scale, the metrics did not follow a consistent latitudinal gradient. For example, the effect of the three-way interaction between depth, latitude, and substrate for invertebrate S was positive in the North region, but negative in the Central region (Table 4 and S3 Table). Our analysis revealed greater complexity in the patterns of both fish and invertebrate diversity at the regional scale compared to the coast-wide scale.

## Discussion

Our study documents diversity patterns and evaluates important abiotic correlates of benthic fish and invertebrate diversity at the scale of a large marine ecosystem. We show that benthic biodiversity in this system does not conform to a simple latitude-diversity gradient, as we expected. Rather, the effect of latitude depends upon the interplay of depth and substrate, and varies depending on year, taxa, and biogeographic region. We also found that fish and invertebrate diversity displayed contrasting responses to depth, which likely reflects key physiological and behavioral differences. Further, by analyzing the influence of latitude, depth, substrate, and year within and across each of the three biogeographical regions, we identified smaller-scale relationships that were obscured at the coast-wide scale.

These findings contribute to a growing body of work that suggests sublittoral to bathyal marine ecosystems do not always closely adhere to a strict latitudinal diversity gradient [81,82]. Although it is possible that our maximum latitudinal range of 15° is insufficient to document a strong and unidirectional relationship with diversity in this system, our range is similar to those used in many other studies of latitudinal gradients of marine biodiversity [17]. Furthermore, latitude was an important predictor of diversity even at the smaller regional scales, suggesting that even modest differences in latitude can explain some of the variability in benthic diversity. Overall, our analysis supports a more nuanced view of the relationships between macroecological diversity patterns, latitude, and other environmental drivers of diversity.

Our findings are in line with research conducted in the North Atlantic, which has documented that patterns of diversity can be better attributed to depth and substrate than latitude [7,28,83]. In addition, depth may act as a proxy for increasing heterogeneity of sediment grain size, a known correlate of infaunal diversity [7]. Etter and Grassle [84], in a study of the western North Atlantic, found that the bathymetric patterns of species diversity were largely attributable to changes in sediment particle diversity, both of which peaked at ~1,500 m and declined thereafter. We have no trawl-specific data on substrate grain size or variability, but our observation of an increase in invertebrate diversity with depth is consistent with this previous work.

Comparing diversity patterns across broad taxonomic groups, we observed divergent responses to depth by fish and invertebrates. Consistent with findings in the Atlantic Ocean, invertebrate diversity displayed a linear increase with depth (Fig 3; [7,28,83]). Deep water bathyal habitats are cold and dark as light only penetrates to a maximum of 1000 m [85]. In deep-water habitats, temperature and productivity are less variable than in nearshore ecosystems [86–88]. Climatic stability has been hypothesized to drive high diversity in terrestrial tropical ecosystems, because it promotes speciation and an increased diversification rate [2,6,89–92]. Thus, deep-water habitats may be analogous to tropical terrestrial ecosystems in that environmental conditions remain stable over time.

In contrast, fish diversity (H′) across the study range did not show a linear relationship with depth but was highest near the continental shelf break (~200 m, Fig 3). Several non-mutually exclusive processes could explain this mid-depth peak. First, the continental shelf zone is highly dynamic, and influenced by upwelling, currents, tides, and internal waves [93]. Along with seasonal changes in water temperature, upwelling brings cold, nutrient-rich water to the surface and enhances primary productivity. High productivity along the shelf break has been shown to fuel aggregations of zooplankton, micronekton, and fish [94] and support distinct assemblages of species that can attain high biomass [95,96], an explanation that is consistent with the long-standing hypothesis that high primary productivity drives the terrestrial latitudinal diversity gradient [2,97]. Second, as a transition zone between shallow shelf lithosphere and the continental slope, the shelf break is associated with high topographical relief and habitat features such as submarine canyons. Thus, mean trawl depth may mask more important predictors of fish diversity, including depth gradient within a single trawl and habitat complexity, both of which would be expected to affect fish diversity [32,98]. Finally, many benthic fish species display ontogenetic shifts in habitat, whereby larval stages reside in estuaries and nearshore habitats and migrate to deeper waters of the continental shelf as adults [99,100]. Thus, the shelf break may be associated with high fish diversity due to its role as an oceanographic, topographic, and biotic transition zone.

The mid-depth peak in fish richness points to one of the limitations of the use of GLMs. Our model-selection and-averaging procedure restricted the candidate set to linear models, as we want to test our hypotheses regarding latitudinal gradients in diversity, but could have potentially obscured non-linear relationships with predictors. For example, invertebrate S and H′ display higher values at the latitudinal extremes of the study area, with lower values in the mid-latitude trawls (see Figs 2 and 3). While some of this variability is accounted for by our co-variates (i.e. depth, substrate, year), the pattern illustrates that a linear latitudinal gradient may not fully describe the complexity of diversity in the CCLME. While outside the scope of this effort, future work should also compare linear to nonlinear models to better elucidate the complex patterns of marine biodiversity.

Our analysis also revealed a measure of scale-dependency in the influence of predictors on biodiversity. Within each biogeographic region, as we found at the coast-wide scale, diversity was influenced by latitude, but did not follow a simple latitudinal gradient. Rather, our results were more consistent with high diversity areas being driven by regional-scale features and biogeographic transition zones. In the CCLME, biogeographic breaks at Cape Mendocino and Point Conception can restrict gene flow within populations [101,102] and may limit dispersal, altering species compositions and ultimately biodiversity [83,103]. Furthermore, the northern and southern areas of our trawl survey, which represent transition zones with neighboring large marine ecosystems, were particularly rich in fish and invertebrates [80]. The area of high biodiversity off the coast of Washington and Northern Oregon is an ecotone between the Gulf of Alaska LME and the CCLME. Here, the Subarctic Current branches, bringing water from the Northern Gulf of Alaska towards the west coast and the CCLME [85]. This delivery of subarctic water is one mechanism for larval dispersal of subarctic species to habitats in the northern CCLME and may explain our observed peak in biodiversity in this region. The California Bight, at the southern-most range of the survey, comprises another area of relatively high biodiversity. This geographic feature is at the southern range limit of many temperate species and the northern range of subtropical species [51]. Thus, the high local species richness (α-diversity) observed at the transition zones in our study may be explained by high regional richness (γ-diversity) in these areas. Similarly, in a variety of other systems, trends in local richness can be at least partially explained by variation in the regional species pool [3,104,105]. Using only a coast-wide scale approach, these regional nuances could be obscured or misinterpreted as a simple large-scale diversity gradient.

In contrast to traditional metrics of species diversity, fish functional trait diversity was related to several environmental gradients at the regional scale, but not at the coast-wide scale. In each biogeographic region, functional diversity was related to latitude, but this relationship was dependent on different environmental factors in each region. This suggests that functional diversity is sensitive to environmental variation in the CCLME, but the correlates of functional diversity are regional.

In some cases, these regional patterns of functional diversity reflect similarities to patterns in traditional metrics of species diversity (e.g. fish S and Q in the Central region were both positively related to latitude and substrate predictors). However, incorporating functional diversity revealed additional relationships between the marine environment and patterns of community structure that were not found using traditional metrics, suggesting that species functional roles do not necessarily follow patterns of taxonomic distinctions among species. This study contributes to a growing body of literature suggesting that fish functional diversity may not vary consistently along continuous gradients, but instead high diversity may be found in regional “hotspots” [44–46].

Inter-annual variation was also an important factor for describing overall biodiversity patterns along the CCLME (Fig 4, S1 and S2 Figs). Year was likely consistently included in our top-ranking models because of strong inter-annual variability of the marine environment along the US west coast. The CCLME is a dynamic ecosystem with divergent climatic conditions occurring on annual timescales. Our study period included a shift from more mild El Niño conditions in the early years of the survey to more turbulent La Niña conditions in the later years [106], which could account for some temporal variation in the data. Larval mobility and settlement in fish and invertebrates may mirror changes in ocean climate conditions [107]; inter-annual variability in these processes may be important in determining biodiversity in any given year. Understanding the drivers of these temporal patterns in biodiversity will be crucial if we are to predict how these areas of high and low biodiversity change annually, over longer time scales, and with a warming climate [33,108].

Our study provides insight into testing macroecological trends in biodiversity using a non-traditional data source. The WCGBTS, a long term, fishery-independent monitoring survey, is meant to collect data to support stock assessments for fisheries management, and basic ecological research is not a primary objective. Despite this, we were able to extract information to answer our fundamental ecological questions on a broad scale. Given the high costs of conducting marine trawl surveys, utilizing previously conducted data to address basic scientific questions is worthwhile and can produce important findings at a low cost, especially when the surveys provide consistent time-series data [109,110]. We propose that these types of datasets represent under-exploited resources for ecologists to conduct basic scientific research. Large-scale monitoring projects, frequently conducted by government institutions, can be used to address broad-scale patterns in species distribution and abundance, and investigate the link between these factors to environmental variability. However, care in interpretation of results should be exercised when using these public datasets, which may give an incomplete picture of biodiversity because the sampling methods are designed to meet other objectives.

In marine ecosystems, complex spatial and temporal patterns of biodiversity have important implications for management, particularly spatial management. Currently, just 3% of US waters are protected by no-take MPAs [111]. Most of these exist in small nearshore pockets that may overlook important diversity elsewhere. For example, of marine reserves in California, offshore soft-bottom habitats are still somewhat underrepresented when compared to rocky habitats [112]. These spatial discrepancies in protection may influence fish biodiversity through removals of commercially important species and invertebrate diversity through habitat impacts [68,113]. Commercial fishing has made a transition to deeper water as shallower, nearshore fisheries have become depleted or closed [86]. We have shown here that benthic fish and invertebrates display different relationships between depth and biodiversity. Deepwater commercial fishing could especially impact invertebrate biodiversity, which we found had highest diversity in deeper waters. Given that fishing practices can influence fish and invertebrate biodiversity in various ways (e.g. [114–118]), future research should explicitly test how fishing pressure may interact with the factors that we found to affect biodiversity in the CCLME.

We used a multi-faceted approach to assess macroecological patterns in benthic biodiversity that combined multiple biodiversity metrics, scales and taxa. As such, we were able to document a high degree of spatial and temporal patterning of biodiversity in the California Current. Our study demonstrates that biodiversity in soft bottom deep water habitats in this system is influenced by latitude, but the effect of latitude is mediated by depth, substrate, and inter-annual variation. Ultimately, the variability in marine benthic biodiversity should be considered in future conservation efforts and for spatial planning.

## Supporting Information

S1 Fig Temporal variation in the relationships among depth and latitude and fish species richness for 2003 through 2010 (A-H). Species richness and color shading scales are not standardized across plots.
(TIF)

S2 Fig Temporal variation in the relationships among depth and latitude and invertebrate species richness for 2004 through 2010 (A-G). Species richness and color shading scales are not standardized across graphs.
(TIF)

S3 Fig Species accumulation curves with 95% confidence intervals for (A) fish and (B) invertebrate species collected in the trawl survey from 2003–2010. (TIF)

S4 Fig Spatial correlograms for (A) fish and (B) invertebrate species collected in the trawl survey from 2003–2010. (TIF)

S1 File Additional information on methods. (PDF)

S1 Table List of families and number of species, of which were identified to the family-level or lower, included in the West Coast Groundfish Bottom Trawl Survey for 2003–2010. Taxa identified to the order-level or higher are not included in this table.
(PDF)

S2 Table Biological traits used in functional diversity metrics for fish. (PDF)

S3 Table Generalized linear model-averaged results for fish richness (S), Shannon diversity (H′) and Rao’s quadratic entropy (Q) and invertebrate S and H′ for the three biogeographic regions, North of Cape Mendocino, the Central Region, and South of Point Conception. The Akaike Information Criterion correction (AICc) was used to rank models and any model that ranked <2 ΔAICc was averaged to obtain final estimates presented. Bolded model terms have a 95% confidence interval (CI) that did not include zero. Relative importance (RI) refers to the proportion of output models that contained the term before the model estimates were averaged.
(PDF)
