# Supplementary figures and images for: Patterns and Variation in Benthic Biodiversity in a Large Marine Ecosystem
Source: PLoS One. 2015 Aug 26;10(8):e0135135. doi: 10.1371/journal.pone.0135135 (PMC4550249; doi:10.1371/journal.pone.0135135)

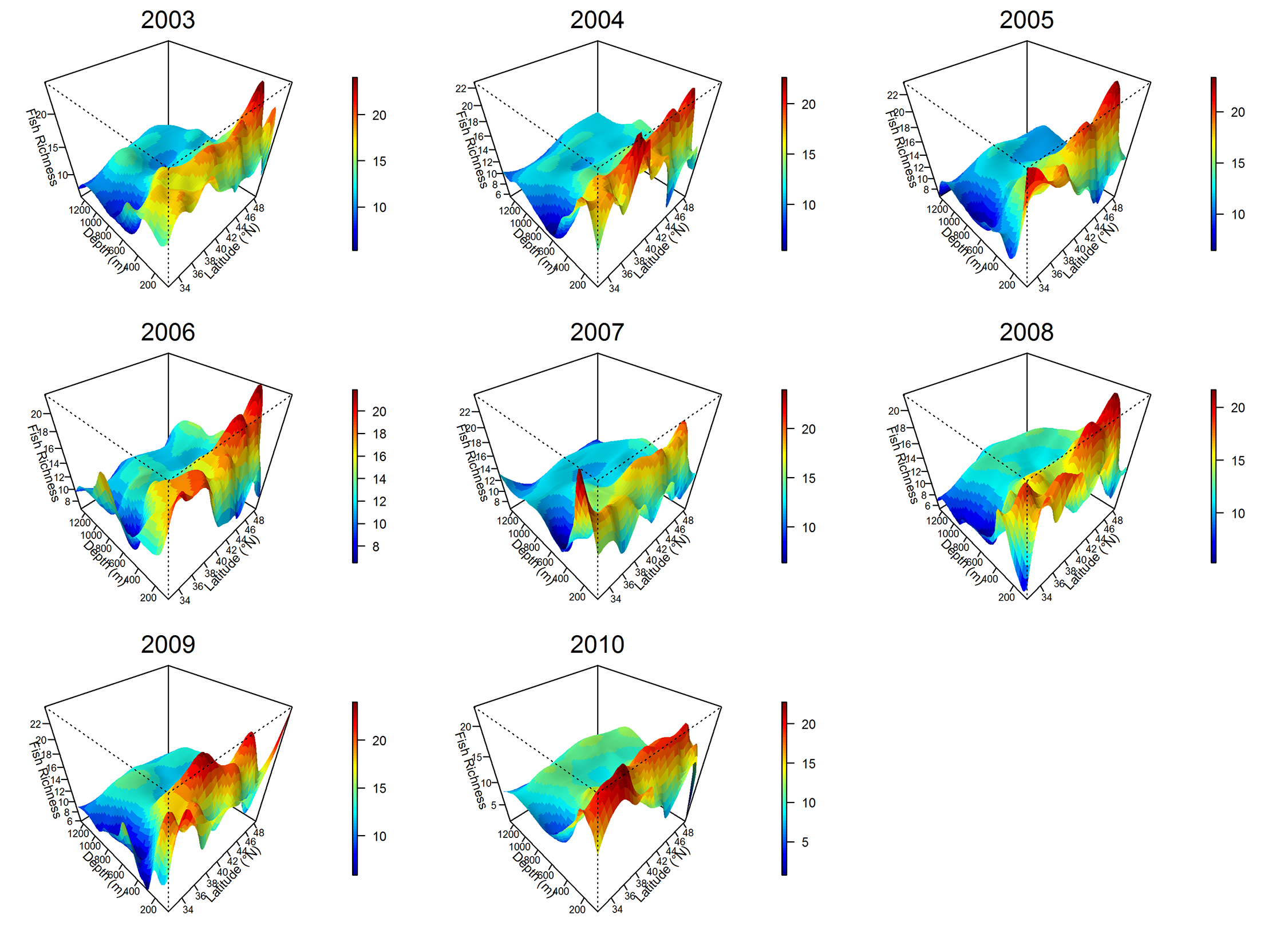

Supplement: S1 Fig — Species richness and color shading scales are not standardized across plots. (TIF) [file pone.0135135.s001.tif]

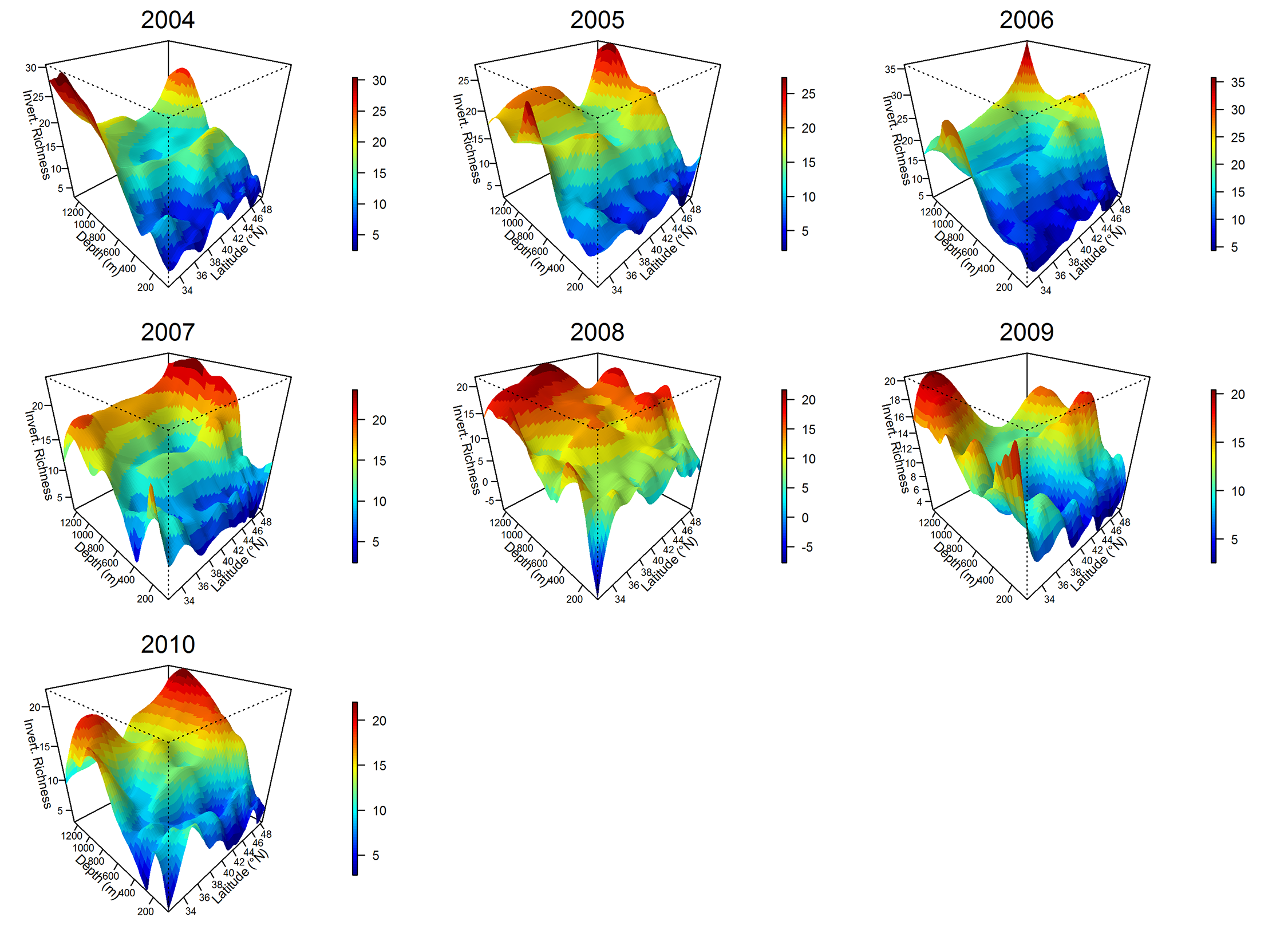

Supplement: S2 Fig — Species richness and color shading scales are not standardized across graphs. (TIF) [file pone.0135135.s002.tif]

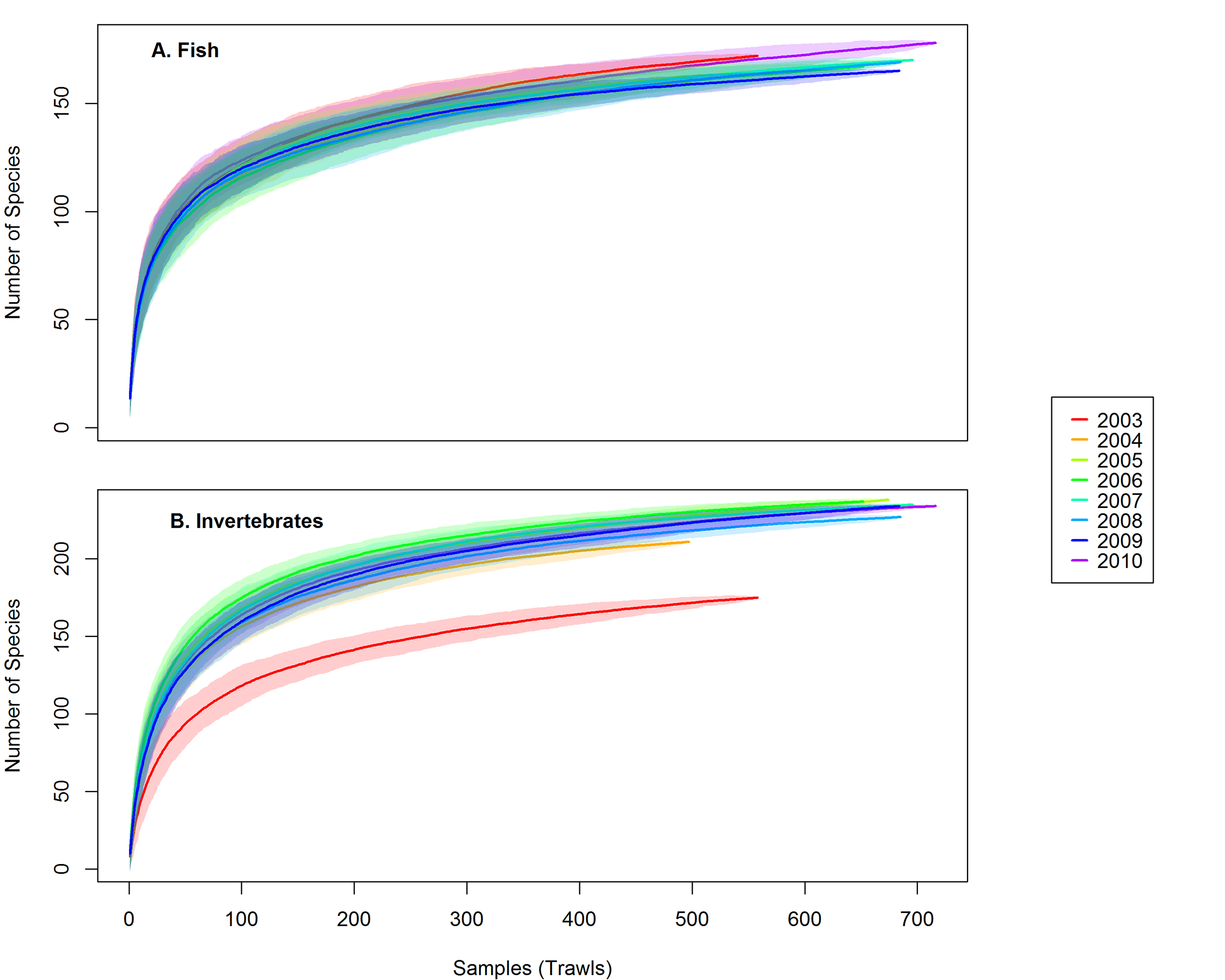

Supplement: S3 Fig — (TIF) [file pone.0135135.s003.tif]

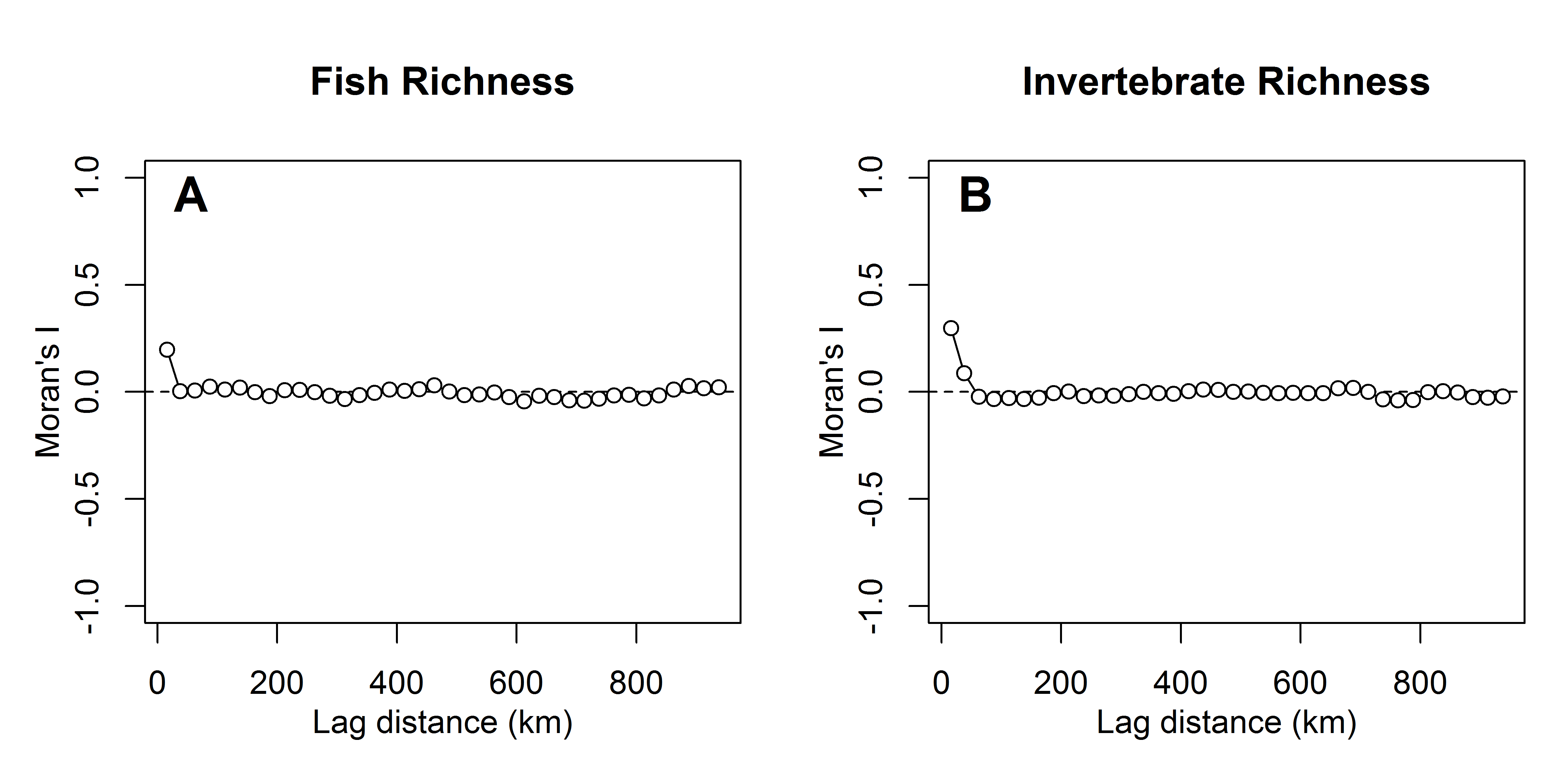

Supplement: S4 Fig — (TIF) [file pone.0135135.s004.tif]
