# Supplementary material for: Patterns and Variation in Benthic Biodiversity in a Large Marine Ecosystem
Source: PLoS One. 2015 Aug 26;10(8):e0135135. doi: 10.1371/journal.pone.0135135 (PMC4550249; doi:10.1371/journal.pone.0135135)
Supplement: S1 File — (PDF) [file pone.0135135.s005.pdf]

S1 File. Additional information on methods.

Data organization

Due to changes in survey protocols through time and differing expertise of scientific crew, there was a degree of inconsistent identification to a taxonomic level, e.g. a species might be identified to the family level or species-level within or across years. We followed these rules to remove or consolidate extraneous taxonomic classifications: 1) all eggs and larval stages were removed, 2) juveniles and adults were combined into one species, 3) if a genus was present, but no individual species from that genus, then the taxon was retained, and likewise for higher order taxonomic classifications, and 4) if genus and a species from that genus both existed, but the total weight of a species was less than the genus, the groups were consolidated into the genus. The last step was a precaution against double counting species and genera, as it was likely there was a higher degree of uncertainty in making identifications to the species level for these taxa. In all, this process would tend to create a bias towards under-counting species. The trawl methodology employed does not provide a standardized measure of pelagic species due to the variability in the time and distance traveled through the water column; therefore, pelagic species were removed. Habitat associations were classified based on a variety of information sources (i.e. FishBase, Encyclopedia of Life, or printed literature).

Functional Diversity

We used the R package *FishBaseR* to download the species-specific trait information [1]. We obtained 18 traits which ranged from trophic, reproductive, morphological, and habitat characteristics. Additionally, we obtained the price category for each species from [2] for a total of 18 traits (S1 Table). For many species, values for a subset of traits were not available in

FishBase. Missing data prevents metric computation as the selected functional diversity metrics are based on inter-species distances calculated using trait values. To overcome this, we assigned missing trait values for species using the median or mode of the genus or family (for continuous or categorical traits, respectively).

Species Accumulation Curves (SACs)

SACs were generated to determine if there was sufficient sampling for both fish and invertebrates and for each year [3]. The SACs for all years combined reached asymptotes in all cases, indicating no need to employ rarefaction to accurately estimate true species richness. However, the SAC for 2003 showed an appreciably lower asymptote than the other years (S3 Fig.). We interpreted this to indicate incomplete sampling and identification of invertebrates to the lowest taxonomic level. For this reason, we excluded 2003 from the analysis of invertebrates. SACs were generated using the random method [4], implemented in the *vegan* package in R 2.12.0 .

Correlograms

As autocorrelation can artificially inflate the potential for Type I error in analyses of spatially explicit data [5], we tested for spatial autocorrelation in the dataset using the *ncf* package in R to produce univariate correlograms [6]. We created a matrix of response measures at each trawl combined with the lag distance to each other trawl point, and tested for correlation between points at each lag distance using Moran's I. We set the increment used to determine distance classes at 50 km, but correlograms were tested at various increments to ensure that patterns were robust to increment length. For each correlogram, the number of pairs in each

distance class and the mean of class were also assessed. The data were resampled 500 times to determine significance values, which were adjusted with the Holm's correction to account for multiple inferences. When the Holm's correction is applied, the results indicate insignificant and low Moran's I values across most spatial lag distances for both fish and invertebrate richness (S4 Fig.).

Spatial Autocorrelation

We analyzed the residuals of the AICc-selected best model to determine if there was any remaining spatial autocorrelation that was unaccounted for in the ΔAICc = 0 model. The residuals of the best model were examined for each year separately using the Moran's I test [5]. We found the spatial autocorrelation was positive and statistically significant only at the lowest distance lag. Moran's I values were near zero, indicating that autocorrelation was not likely to substantially affect our conclusions. Further, autocorrelation declined to zero after the first 25 km lag distance. Thus, we determined the model terms sufficiently accounted for autocorrelation at the scale of our inference and we did not need to further build in a correlation structure.

References

1. Cornejo-Donoso J. FishBaseR: Scrape FishBase database (http://www.fishbase.org) for data. R package version 0.9.1. 2011.
2. Sumaila UR, Marsden AD, Watson R, Pauly D. A Global Ex-vessel Fish Price Database: Construction and Applications. J Bioeconomics. 2007;9: 39–51. doi:10.1007/s10818-007-9015-4
3. Bonar S, Fehmi J, Mercado-Silva N. An overview of sampling issues in species diversity and abundance surveys. In: Magurran AE, McGill BJ, editors. Biological Diversity: Frontiers in Measurement and Assessment. Oxford, U.K.: Oxford University Press; 2011. p. 345.
4. Gotelli NJ, Colwell RK. Quantifying biodiversity: procedures and pitfalls in the

measurement and comparison of species richness. Ecol Lett. 2001;4: 379–391. doi:10.1046/j.1461-0248.2001.00230.x
5. Fortin MJ, Dale MRT. Spatial Analysis: A Guide for Ecologists. Cambridge, UK: Cambridge University Press; 2005.
6. Bjornstad ON. Ncf: spatial nonparametric covariance functions [Internet]. 2009. Available: http://CRAN.R-project.org/package=ncf
