# Supplementary material for: Patterns and Variation in Benthic Biodiversity in a Large Marine Ecosystem
Source: PLoS One. 2015 Aug 26;10(8):e0135135. doi: 10.1371/journal.pone.0135135 (PMC4550249; doi:10.1371/journal.pone.0135135)
Supplement: S1 Table — Taxa identified to the order-level or higher are not included in this table. (PDF) [file pone.0135135.s006.pdf]

S1 Table. List of families and number of species, of which were identified to the family-level or lower, included in the West Coast Groundfish Bottom Trawl Survey for 2003-2010.

Family	Number of species	Family	Number of species
Acanthogorgiidae	1	Cottidae	24
Actiniidae	1	Cranchiidae	3
Actinostolidae	2	Cryptacanthodidae	2
Agonidae	8	Ctenodiscidae	1
Alepocephalidae	3	Cucumariidae	1
Ammodytidae	1	Cynoglossidae	1
Amphiuridae	1	Discodorididae	1
Anarhichadidae	1	Dorididae	2
Anoplopomatidae	1	Echinasteridae	2
Antedonidae	1	Elipiidae	1
Aphroditidae	1	Embiotocidae	10
Argentinidae	1	Epialtidae	1
Arhynchobatidae	4	Etmopteridae	2
Arminidae	1	Etmopteridae/Scyliorhinidae	2
Asteriidae	9	Euryalidae	1
Asterinidae	1	Farreidae	1
Asteronychidae	2	Fasciolariidae	1
Asteropseidae	1	Fissurellidae	1
Astropectinidae	7	Flabellidae	1
Bathymasteridae	1	Gonatidae	3
Batrachoididae	1	Goniasteridae	6

Benthopectinidae	2	Gorgonocephalidae	1
Bolitaenidae	1	Grimpoteuthidae	1
Brisingidae	1	Halipteridae	1
Brissidae	1	Hexagrammidae	4
Buccinidae	4	Hexanchidae	1
Bursidae	1	Hiatellidae	1
Bythitidae	2	Hippolytidae	1
Callianassidae	1	Histioteuthidae	2
Calliostomatidae	1	Hormathiidae	3
Calliotropidae	1	Hyalonematidae	1
Cancellariidae	1	Isididae	2
Caryophylliidae	1	Laetmogonidae	1
Caulophrynidae	1	Lepadidae	2
Chimaeridae	1	Limidae	1
Chiroteuthidae	1	Liparidae	2
Cladopathidae	1	Liparidinae	13
Clinidae	1	Lithodidae	2
Cochlespiridae	1	Luidiidae	2
Coenobitidae	1	Majidae	2
Merlucciidae	3	Rajidae	4
Molpadiidae	1	Ranellidae	1
Moridae	1	Rhinobatidae	1
Muricidae	1	Rhinochimaeridae	1
Myliobatidae	1	Rhodaliidae	1
Mytilidae	1	Rossellidae	1
Myxasteridae	1	Salmonidae	1

Myxinidae	2	Sareptidae	1
Naticidae	4	Schizasteridae	2
Nematocarcinidae	1	Sciaenidae	2
Neomeniidae	1	Scorpaenidae	55
Nephtyidae	1	Scyliorhinidae	2
Nereididae	1	Sepiolidae	1
Nettastomatidae	2	Serranidae	1
Octopodidae	5	Solasteridae	10
Octopoteuthidae	1	Solemyidae	2
Ommastrephidae	1	Somnosidae	1
Onychoteuthidae	2	Spatangidae	1
Ophiacanthidae	2	Squalidae	1
Ophiactidae	1	Squatinidae	1
Ophidiidae	3	Stichaeidae	1
Ophiolepididae	2	Stichopodidae	3
Ophiomyxidae	2	Stromateidae	1
Ophiuridae	3	Strongylocentrotidae	2
Opisthoteuthidae	1	Styelidae	1
Oregoniidae	1	Stylasteridae	1
Osmeridae	4	Suberitidae	1
Pandalidae	1	Synallactidae	2
Paralichthyidae	3	Synodontidae	2
Pasiphaeidae	51	Teredinidae	1
Pectinidae	4	Toxopneustidae	1
Pedicellasteridae	2	Triakidae	3
Pennatulidae	1	Trichiuridae	3

Percichthyidae	1	Trichodontidae	1
Petromyzontidae	1	Triglidae	2
Philinoidea	1	Tritoniidae	2
Phyllophoridae	1	Trochidae	1
Pleurobranchaiedae	1	Uranoscopidae	1
Pleuronectidae	20	Vampyroteuthidae	1
Poraniidae	3	Velutinidae	1
Pseudarchasteridae	6	Verticordiidae	1
Psolidae	2	Vesicomyidae	1
Psychrolutidae	3	Virgulariidae	22
Pterasteridae	5	Zoarcidae	12
Ptychatractidae	1	Zoroasteridae	3
Pyuridae	1	**Total Species**	**500**

Taxa identified to the order-level or higher are not included in this table.
