# Supplementary material for: Patterns and Variation in Benthic Biodiversity in a Large Marine Ecosystem
Source: PLoS One. 2015 Aug 26;10(8):e0135135. doi: 10.1371/journal.pone.0135135 (PMC4550249; doi:10.1371/journal.pone.0135135)
Supplement: S2 Table — (PDF) [file pone.0135135.s007.pdf]

S2 Table. Biological traits used in functional diversity metrics for fish.

Functional Trait	Modality	Type of data
Maximum length	cm	Continuous
L_∞ - asymptotic length at which growth is zero	From von Bertalanffy growth equation	Continuous
K - growth rate	From von Bertalanffy growth equation	Continuous
Trophic level	Calculated from diet composition in FishBase	Continuous
Temperature range – minimum		Continuous
Temperature range – maximum		Continuous
Depth range – minimum	Meters	Continuous
Depth range – maximum	Meters	Continuous
Main diet item	Zoobenthos, Zooplankton, Nekton, Detritus, Plants, Others	Categorical
Trophic level of the diet	Calculated from diet composition in FishBase	Continuous
Age at maturity – minimum	Years	Continuous
Age at maturity – maximum	Years	Continuous
Reproductive mode	Dioecism, Parthenogenesis, Protandry, Protogyny, True Hermaphroditism	Categorical
Larval duration	Days to settlement	Continuous
Habitat association	Bathydemersal, Bathypelagic, Benthopelagic, Demersal, Pelagic-neritic, Pelagic-oceanic, Reef associated	Categorical

Price category – based on ex-vessel prices in 2000, US dollars (Sumaila et al. 2007)	Very high, high, medium, low, not marketed/unknown, unknown	Categorical
a – coefficient, varies between species	From length-weight equation for fish	Continuous
b – >3 = greater girth, <3 = more streamlined	From length-weight equation for fish	Continuous
