# Supplementary material for: Patterns and Variation in Benthic Biodiversity in a Large Marine Ecosystem
Source: PLoS One. 2015 Aug 26;10(8):e0135135. doi: 10.1371/journal.pone.0135135 (PMC4550249; doi:10.1371/journal.pone.0135135)
Supplement: S3 Table — The Akaike Information Criterion correction (AICc) was used to rank models and any model that ranked <2 ΔAICc was averaged to obtain final estimates presented. Bolded model terms have a 95% confidence interval (CI) that did not include zero. Relative importance (RI) refers to the proportion of output models that contained the term before the model estimates were averaged. (PDF) [file pone.0135135.s008.pdf]

S3 Table. Generalized linear model-averaged results for fish richness (S), Shannon diversity (H′) and Rao's quadratic entropy (Q) and invertebrate S and H′ for the three biogeographic regions, North of Cape Mendocino, the Central Region, and South of Point Conception.

Taxa Metric Region	Variable	Estimate	Adjusted SE	Lower CI	Upper CI	RI
**Fish S**						
**North**	(Intercept)	23.13	40.42	-56.09	102.36	.
	Depth	-0.0218	0.0178	-0.0567	0.0131	1.00
	Year	-0.0124	0.0216	-0.0548	0.0300	0.82
	**Depth·Year**	**1.44E-05**	**7.29E-06**	**7.49E-08**	**2.87E-05**	**0.75**
	Substrate	-270.25	672.80	-1588.92	1048.42	0.54
	Latitude	-0.5572	1.3759	-3.2539	2.1395	0.36
	Latitude·Substrate	18.44	21.33	-23.36	60.23	0.18
	Latitude·Year	0.0011	0.0010	-0.0009	0.0030	0.09
	Substrate·Year	0.4476	0.4833	-0.4997	1.3949	0.16
	**Latitude·Substrate·Year**	**-0.0177**	**0.0082**	**-0.0337**	**-0.0018**	**0.09**
**Central**	(Intercept)	-249.07	258.90	-756.51	258.37	.
	Depth	0.3957	0.4654	-0.5164	1.3078	1.00
	Latitude	7.7858	6.9481	-5.8322	21.4038	1.00
	Substrate	-13.39	24.40	-61.21	34.43	1.00
	Year	0.1263	0.1290	-0.1266	0.3792	1.00
	Depth·Latitude	-0.0116	0.0125	-0.0361	0.0128	1.00
	Depth·Year	-0.0002	0.0002	-0.0007	0.0003	1.00
	**Latitude·Substrate**	**0.0598**	**0.0224**	**0.0159**	**0.1036**	**1.00**
	Latitude·Year	-0.0054	0.0029	-0.0111	0.0004	0.73

	Depth·Latitude·Year	9.74E-06	5.13E-06	-3.07E-07	1.98E-05	0.60
	Substrate·Year	0.0181	0.0157	-0.0127	0.0489	0.31
	Depth·Substrate	-0.0001	0.0001	-0.0003	0.0001	0.12
**South**	(Intercept)	703.64	869.58	-1000.71	2407.98	.
	**Depth**	**3.8331**	**1.6981**	**0.5049**	**7.1613**	**1.00**
	Latitude	-21.4411	25.8089	-72.0255	29.1434	1.00
	Substrate	-3403.92	3074.86	-9430.53	2622.69	1.00
	Year	-0.3525	0.4334	-1.2019	0.4970	1.00
	**Depth·Latitude**	**-0.1131**	**0.0510**	**-0.2129**	**-0.0132**	**1.00**
	**Depth·Substrate**	**-0.0250**	**0.0107**	**-0.0459**	**-0.0042**	**1.00**
	**Depth·Year**	**-0.0019**	**0.0008**	**-0.0036**	**-0.0002**	**1.00**
	Latitude·Substrate	101.54	91.77	-78.32	281.40	1.00
	Latitude·Year	0.0108	0.0129	-0.0144	0.0360	1.00
	**Substrate·Year**	**2.5907**	**1.1252**	**0.3853**	**4.7960**	**0.65**
	**Depth·Latitude·Substrate**	**0.0008**	**0.0003**	**0.0001**	**0.0014**	**1.00**
	**Depth·Latitude·Year**	**5.60E-05**	**2.54E-05**	**6.20E-06**	**1.06E-04**	**1.00**
	**Latitude·Substrate·Year**	**-0.0773**	**0.0336**	**-0.1432**	**-0.0114**	**0.65**
**Fish H′**						
**North**	(Intercept)	21.86	44.28	-64.93	108.65	.
	Depth	0.0024	0.0108	-0.0187	0.0235	1.00
	Latitude	-0.1479	0.9719	-2.0529	1.7571	1.00
	Year	-0.0101	0.0221	-0.0534	0.0331	1.00
	Depth·Latitude	1.507E-05	9.372E-06	-3.298E-06	3.344E-05	0.62
	Substrate	0.0342	0.0366	-0.0374	0.1059	0.25
	Depth·Year	-7.402E-06	9.457E-06	-2.594E-05	1.113E-05	0.22
	Latitude·Year	0.0007	0.0013	-0.0019	0.0033	0.11
**Central**	(Intercept)	174.16	293.04	-400.19	748.51	.

	Depth	0.1955	0.5396	-0.8621	1.2531	1.00
	Latitude	-3.0614	7.8550	-18.4570	12.3342	1.00
	Substrate	17.67	37.06	-54.96	90.30	1.00
	Year	-0.0854	0.1460	-0.3716	0.2009	1.00
	Depth·Year	-0.0001	0.0003	-0.0006	0.0004	1.00
	Latitude·Substrate	0.0610	0.0333	-0.0042	0.1262	0.83
	Latitude·Year	0.0019	0.0043	-0.0066	0.0104	0.78
	Depth·Latitude	-0.0276	0.0144	-0.0558	0.0007	0.28
	Depth·Latitude·Year	1.375E-05	7.186E-06	-3.385E-07	2.783E-05	0.28
	Substrate·Year	-0.0242	0.0221	-0.0675	0.0191	0.40
**South**	(Intercept)	-166.73	1799.29	-3693.28	3359.82	.
	Depth	4.6814	3.5659	-2.3076	11.6704	1.00
	Latitude	6.2447	53.5876	-98.7850	111.2744	1.00
	Substrate	-276.27	1408.63	-3037.14	2484.60	1.00
	Year	0.0821	0.8967	-1.6753	1.8396	1.00
	Depth·Latitude	-0.1407	0.1070	-0.3504	0.0690	1.00
	Depth·Substrate	-0.0057	0.0573	-0.1180	0.1067	1.00
	**Depth·Year**	**-0.0031**	**0.0014**	**-0.0058**	**-0.0004**	**0.76**
	Latitude·Substrate	6.8067	42.0300	-75.5705	89.1840	1.00
	Latitude·Year	-0.0031	0.0267	-0.0554	0.0493	1.00
	Substrate·Year	0.2014	0.8547	-1.4737	1.8766	0.66
	**Depth·Latitude·Year**	**0.0001**	**4.104E-05**	**1.179E-05**	**0.0002**	**0.76**
	Latitude·Substrate·Year	-0.0324	0.0584	-0.1467	0.0820	0.10
	Depth·Substrate·Year	0.0000	0.0001	-0.0001	0.0002	0.09
	Depth·Latitude·Substrate	0.0002	0.0005	-0.0009	0.0012	0.09
**Fish Q**						
**North**	(Intercept)	-2.90	4.28	-11.3	5.48	.

	Depth	0.0066	0.00822	-0.00952	0.0227	1
	Latitude	0.078885	0.0952	-0.108	0.265	1
	Substrate	-0.28862	0.472	-1.21399	0.637	1
	Year	0.001451	0.00213	-0.00273	0.00563	1
	Depth·Latitude	-0.00029	0.000159	-0.0006	2.24E-05	0.51
	**Depth·Year**	**-7.36E-06**	**2.76E-06**	**-1.28E-05**	**-1.94E-06**	0.45
	**Latitude·Substrate**	**0.000831**	**0.000339**	**0.000166**	**0.001496**	1
	Latitude·Year	-7.58E-05	3.97E-05	-0.00015	1.93E-06	0.52
	**Depth·Latitude·Year**	**1.65E-07**	**6.21E-08**	**4.30E-08**	**2.87E-07**	0.45
	Substrate·Year	0.000335	0.000279	-0.00021	0.000881	0.37
	Depth·Substrate	3.18E-06	2.77E-06	-2.24E-06	8.61E-06	0.36
**Central**	(Intercept)	6.79	4.68	-2.38	16.0	.
	Depth	0.00295	0.00776	-0.0123	0.0182	1
	Latitude	-0.165	0.126	-0.412	0.0818	1
	Substrate	0.167	0.395	-0.608	0.941	0.60
	Year	-0.00338	0.00233	-0.00795	0.00119	1
	Depth·Latitude	-9.13E-05	0.000212	-0.00051	0.000325	1
	**Latitude·Substrate**	**-0.00229**	**0.00113**	**-0.0045**	**-8.71E-05**	**0.60**
	Latitude·Year	8.24E-05	6.28E-05	-4.07E-05	0.000206	1
	Depth·Year	-2.32E-06	4.67E-06	-1.15E-05	6.84E-06	1
	Depth·Latitude·Year	1.81E-07	1.43E-07	-9.88E-08	4.61E-07	0.25
	Substrate·Year	-0.00019	0.000378	-0.00093	0.000553	0.14
**South**	(Intercept)	3.21	7.42	-11.3	17.7	.
	Depth	0.000175	0.000238	-0.00029	0.000641	1
	Latitude	-0.0743	0.220	-0.506	0.358	1
	Substrate	-0.297	0.581	-1.43	0.841	1
	Year	-0.00161	0.00370	-0.00885	0.00564	1

	**Depth·Latitude**	**-6.18E-06**	**1.27E-06**	**-8.66E-06**	**-3.70E-06**	**1**
	**Depth·Substrate**	**8.54E-06**	**2.96E-06**	**2.74E-06**	**1.43E-05**	**1**
	Substrate·Year	0.000436	0.000356	-0.00026	0.00113	0.33
	Latitude·Substrate	0.000922	0.000176	-0.0002	0.000491	0.12
	Latitude·Year	0.000146	0.00172	-0.00245	0.00430	0.26
	Depth·Year	1.42E-07	3.12E-07	-4.70E-07	7.54E-07	0.12
**Invert S**						
**North**	**(Intercept)**	**550.85**	**174.83**	**208.20**	**893.51**	**.**
	Depth	0.0043	0.0157	-0.0264	0.0351	1.00
	**Latitude**	**-11.33**	**3.89**	**-18.96**	**-3.71**	**1.00**
	Substrate	6.7880	22.3410	-36.9996	50.5756	1.00
	**Year**	**-0.2734**	**0.0871**	**-0.4442**	**-0.1027**	**1.00**
	**Depth·Latitude**	**4.19E-05**	**1.06E-05**	**2.10E-05**	**0.0001**	**1.00**
	**Depth·Substrate**	**-0.0235**	**0.0087**	**-0.0406**	**-0.0064**	**1.00**
	**Latitude·Substrate**	**-0.2351**	**0.0525**	**-0.3381**	**-0.1322**	**1.00**
	**Latitude·Year**	**0.0056**	**0.0019**	**0.0018**	**0.0094**	**1.00**
	**Depth·Latitude·Substrate**	**0.0005**	**0.0002**	**0.0002**	**0.0009**	**1.00**
	Depth·Year	-1.00E-05	1.27E-05	-3.49E-05	1.48E-05	0.26
	Substrate·Year	0.0092	0.0226	-0.0351	0.0534	0.21
**Central**	(Intercept)	60.50	184.95	-301.99	422.99	.
	**Depth**	**0.1638**	**0.0275**	**0.1099**	**0.2178**	**1.00**
	Latitude	-2.8102	4.9465	-12.5051	6.8847	1.00
	Substrate	-38.06	50.09	-136.24	60.12	1.00
	Year	-0.0287	0.0921	-0.2093	0.1519	1.00
	Depth·Latitude	1.64E-05	1.49E-05	-1.29E-05	4.57E-05	0.45
	Depth·Substrate	-0.0101	0.0585	-0.1247	0.1046	0.45
	**Depth·Year**	**-0.0001**	**0.0000**	**-0.0001**	**-0.0001**	**1.00**

	Latitude·Substrate	0.0961	0.0809	-0.0626	0.2547	0.58
	Substrate·Year	0.0286	0.0263	-0.0230	0.0802	0.63
	**Depth·Latitude·Substrate**	**-0.0002**	**0.0001**	**-0.0003**	**0.0000**	**0.26**
	Latitude·Year	0.0035	0.0028	-0.0020	0.0090	0.39
	Depth·Substrate·Year	0.0001	0.0001	-0.0001	0.0002	0.06
**South**	(Intercept)	-4.8516	393.80	-776.69	766.99	.
	**Depth**	**0.2397**	**0.0463**	**0.1489**	**0.3304**	**1.00**
	Latitude	-1.8936	11.5912	-24.6120	20.8247	1.00
	Substrate	-3.2300	46.91	-95.17	88.71	1.00
	Year	0.0032	0.1962	-0.3815	0.3878	1.00
	**Depth·Latitude**	**-0.0003**	**0.0001**	**-0.0005**	**-0.0001**	**1.00**
	Depth·Substrate	-0.0380	0.0998	-0.2337	0.1577	1.00
	**Depth·Year**	**-0.0001**	**2.29E-05**	**-0.0002**	**-0.0001**	**1.00**
	Latitude·Substrate	0.2468	0.2063	-0.1576	0.6512	1.00
	Depth·Latitude·Substrate	0.0006	0.0005	-0.0004	0.0017	0.26
	Substrate·Year	-0.0093	0.0434	-0.0942	0.0757	0.28
	**Depth·Substrate·Year**	**0.0001**	**0.0001**	**-3.79E-05**	**0.0003**	**0.14**
	Latitude·Year	0.0072	0.0144	-0.0210	0.0355	0.13
**Invert H′**						
**North**	(Intercept)	422.98	408.55	-377.77	1223.73	.
	Depth	-0.6880	0.7748	-2.2065	0.8305	1.00
	Latitude	-8.7827	9.0995	-26.6174	9.0521	1.00
	Substrate	-305.96	1027.43	-2319.69	1707.77	1.00
	Year	-0.2102	0.2036	-0.6093	0.1888	1.00
	Depth·Latitude	0.0185	0.0181	-0.0169	0.0540	0.71
	Depth·Year	0.0003	0.0004	-0.0004	0.0011	1.00
	Latitude·Substrate	6.8243	22.8723	-38.0045	51.6532	1.00

	Latitude·Year	0.0064	0.0041	-0.0017	0.0145	0.68
	Depth·Latitude·Year	-1.43E-05	7.33E-06	-2.86E-05	1.16E-07	0.46
	Substrate·Year	1.3174	0.8351	-0.3193	2.9542	0.12
	Latitude·Substrate·Year	-0.0294	0.0185	-0.0657	0.0070	0.12
	Depth·Substrate	-0.0001	0.0003	-0.0007	0.0006	0.09
**Central**	**(Intercept)**	**-1556.37**	**459.88**	**-2457.72**	**-655.02**	**.**
	Depth	1.5369	0.8174	-0.0652	3.1391	1.00
	**Latitude**	**42.53**	**12.32**	**18.39**	**66.68**	**1.00**
	Substrate	5488.76	4836.87	-3991.34	14968.85	1.00
	**Year**	**0.7770**	**0.2291**	**0.3279**	**1.2260**	**1.00**
	**Depth·Latitude**	**-0.0441**	**0.0220**	**-0.0872**	**-0.0009**	**1.00**
	Depth·Substrate	-7.1654	6.3010	-19.5151	5.1843	1.00
	**Depth·Year**	**-0.0008**	**0.0004**	**-0.0016**	**0.0000**	**1.00**
	Latitude·Substrate	-164.41	133.17	-425.42	96.60	1.00
	**Latitude·Year**	**-0.0212**	**0.0061**	**-0.0332**	**-0.0092**	**1.00**
	Substrate·Year	-2.7395	2.4107	-7.4644	1.9854	1.00
	**Depth·Latitude·Substrate**	**0.3023**	**0.1254**	**0.0565**	**0.5481**	**0.71**
	**Depth·Latitude·Year**	**2.20E-05**	**1.10E-05**	**4.59E-07**	**4.34E-05**	**1.00**
	Depth·Substrate·Year	0.0036	0.0031	-0.0026	0.0097	1.00
	**Latitude·Substrate·Year**	**0.1148**	**0.0491**	**0.0186**	**0.2109**	**0.71**
	**Depth·Latitude·Substrate·Year**	**-0.0002**	**0.0001**	**-0.0003**	**0.0000**	**0.71**
**South**	(Intercept)	2497.54	1418.81	-283.27	5278.35	.
	Depth	0.5553	1.7537	-2.8818	3.9925	1.00
	Latitude	-76.55	41.91	-158.69	5.59	1.00
	Substrate	-0.6109	1.9500	-4.4329	3.2111	1.00
	Year	-1.2458	0.7070	-2.6315	0.1399	1.00
	Depth·Latitude	-0.0143	0.0523	-0.1168	0.0883	1.00

	**Depth·Substrate**	**0.0008**	**0.0002**	**0.0004**	**0.0013**	**1.00**
	Depth·Year	-0.0004	0.0010	-0.0023	0.0016	0.77
	Latitude·Year	0.0382	0.0209	-0.0027	0.0791	1.00
	Depth·Latitude·Year	3.59E-05	4.99E-05	-0.0001	0.0001	0.19
	Latitude·Substrate	0.0611	0.1319	-0.1975	0.3197	0.16

The Akaike Information Criterion correction (AICc) was used to rank models and any model that ranked <2 ΔAICc was averaged to obtain final estimates presented. Bolded model terms have a 95% confidence interval (CI) that does not include zero. Relative importance (RI) refers to the proportion of output models that contained the term before the model estimates were averaged.
